# Pyruvate kinase M2 regulates homologous recombination-mediated DNA double-strand break repair

**DOI:** 10.1038/s41422-018-0086-7

**Published:** 2018-10-08

**Authors:** Steven T. Sizemore, Manchao Zhang, Ju Hwan Cho, Gina M. Sizemore, Brian Hurwitz, Balveen Kaur, Norman L. Lehman, Michael C. Ostrowski, Pierre A. Robe, Weili Miao, Yinsheng Wang, Arnab Chakravarti, Fen Xia

**Affiliations:** 1 Department of Radiation Oncology, Arthur G James Comprehensive Cancer Center and Richard L. Solove Research Institute, The Ohio State University Medical Center, Columbus, OH 43210 USA; 2 Department of Radiation Oncology, University of Arkansas for Medical Sciences, Little Rock, AR 72205 USA; 3 Department of Cancer Biology & Genetics, Arthur G James Comprehensive Cancer Center and Richard L. Solove Research Institute, The Ohio State University Medical Center, Columbus, OH 43210 USA; 4 Department of Neurological Surgery, Arthur G James Comprehensive Cancer Center and Richard L. Solove Research Institute, The Ohio State University Medical Center, Columbus, OH 43210 USA; 5 Department of Pathology, Arthur G James Comprehensive Cancer Center and Richard L. Solove Research Institute, The Ohio State University Medical Center, Columbus, OH 43210 USA; 6 Department of Neurology and Neurosurgery, Rudolf Magnus Brain Institute, University Medical Center of Utrecht, Utrecht, The Netherlands; 7 Departments of Neurosurgery and Human Genetics, University of Liege, Liege, Belgium; 8 Department of Chemistry, University of California, Riverside, CA 92521 USA

## Abstract

Resistance to genotoxic therapies is a primary cause of treatment failure and tumor recurrence. The underlying mechanisms that activate the DNA damage response (DDR) and allow cancer cells to escape the lethal effects of genotoxic therapies remain unclear. Here, we uncover an unexpected mechanism through which pyruvate kinase M2 (PKM2), the highly expressed PK isoform in cancer cells and a master regulator of cancer metabolic reprogramming, integrates with the DDR to directly promote DNA double-strand break (DSB) repair. In response to ionizing radiation and oxidative stress, ATM phosphorylates PKM2 at T328 resulting in its nuclear accumulation. pT328-PKM2 is required and sufficient to promote homologous recombination (HR)-mediated DNA DSB repair through phosphorylation of CtBP-interacting protein (CtIP) on T126 to increase CtIP’s recruitment at DSBs and resection of DNA ends. Disruption of the ATM-PKM2-CtIP axis sensitizes cancer cells to a variety of DNA-damaging agents and PARP1 inhibition. Furthermore, increased nuclear pT328-PKM2 level is associated with significantly worse survival in glioblastoma patients. Combined, these data advocate the use of PKM2-targeting strategies as a means to not only disrupt cancer metabolism but also inhibit an important mechanism of resistance to genotoxic therapies.

## Introduction

Resistance to genotoxic therapies, such as radiation and DNA-damaging chemotherapeutics, is the primary cause of treatment failure for many cancers. Double-strand breaks (DSBs) account for the majority of the cytotoxicity associated with these treatments and cellular response to genotoxic stress is ultimately determined by repair of these lethal lesions. There are two primary pathways, non-homologous end-joining (NHEJ) and homologous recombination (HR), to repair DNA DSBs. NHEJ takes place during all phases of the cell cycle and is the predominant repair pathway during the G1/G0 phase while HR repair primarily occurs during S phase.^1,2^

The serine/threonine kinase ataxia telangiectasia mutated (ATM) is a key protein kinase that regulates multiple DDR processes including DNA repair through the NHEJ and HR pathways.^3^ While both the NHEJ and HR pathways are involved in cancer resistance to genotoxic therapies, the HR repair pathway is particularly critical in highly proliferative cancer cells. HR-mediated repair utilizes intact homologous DNA sequences as templates to repair DSBs with high fidelity. CtBP-interacting protein (CtIP) is a key rate-limiting component of HR repair that interacts with the Mre11/Rad50/Nbs1 (MRN) complex to promote DSB end-resection, generation of ssDNA tails, and initiation of DSB repair.^4^ While ATM and CtIP are indisputably important mediators of cancer resistance to genotoxic agents, efforts to reduce cancer cell resistance to therapy via directly targeting these molecules are inherently limited given their essential functions in normal cells. Identification of ATM substrates and/or CtIP effectors that are vital to DNA DSB repair in cancer cells but are dispensable to repair in normal cells could provide essential tools to combat treatment resistance.

Metabolic reprogramming, including aerobic glycolysis, known as the Warburg effect, is one of the most obvious and universal differences between cancer cells and their cognate normal cell of origin. While most of the key enzymes involved in glycolysis are shared between cancer and normal cells, overexpression of pyruvate kinase M2 (PKM2) in cancer cells drives the Warburg effect.^5^ A growing body of evidence suggests that PKM2 supports cancer cell metabolism and growth not only through its pyruvate kinase activity in the cytosol, but also through its more recently discovered nuclear function as transcriptional coactivator. Nuclear PKM2 regulates expression of genes encoding glucose transporter 1 (*GLUT1/SLC2A1)* and lactate dehydrogenase A (*LDHA)*^5^ in a positive feed-forward manner to further support glycolytic metabolism. A number of post-translational modifications, including phosphorylation by ERK1/2,^5,6^ have been identified to regulate PKM2’s cytoplasmic/nuclear distribution.

Emerging evidence indicates that coordination between glucose metabolism and DNA DSB repair is important for sustained cancer cell proliferation and survival from intracellular oxidative stress, and genotoxic treatment.^7,8^ However, the underlying mechanisms that enable crosstalk between these processes remain to be elucidated. In this study, we demonstrate a novel mechanism through which PKM2 directly regulates DSB repair to provide resistance to DNA damage. PKM2 phosphorylates CtIP at T126 to activate CtIP’s end-resection function and increase HR-mediated DSB repair. ATM is identified as the upstream mediator that phosphorylates nuclear PKM2 at T328 following DNA damage. Phosphorylation of T328 in the nucleus is required and sufficient to increase PKM2’s nuclear accumulation and its interaction with and phosphorylation of CtIP. Furthermore, the nuclear level of T328-phosphorylated PKM2, a readout of ATM-PKM2-CtIP pathway activity, is prognostic of poor overall survival in glioblastoma patients. Our findings reveal that PKM2, a key controller of cancer metabolic reprogramming, directly regulates DNA repair and advocate PKM2-targeted therapies to combat treatment resistance and improve cancer patient outcomes.

## Results

### PKM2 is a critical mediator of cellular resistance to DNA-damaging treatment

**Fig. 1:**
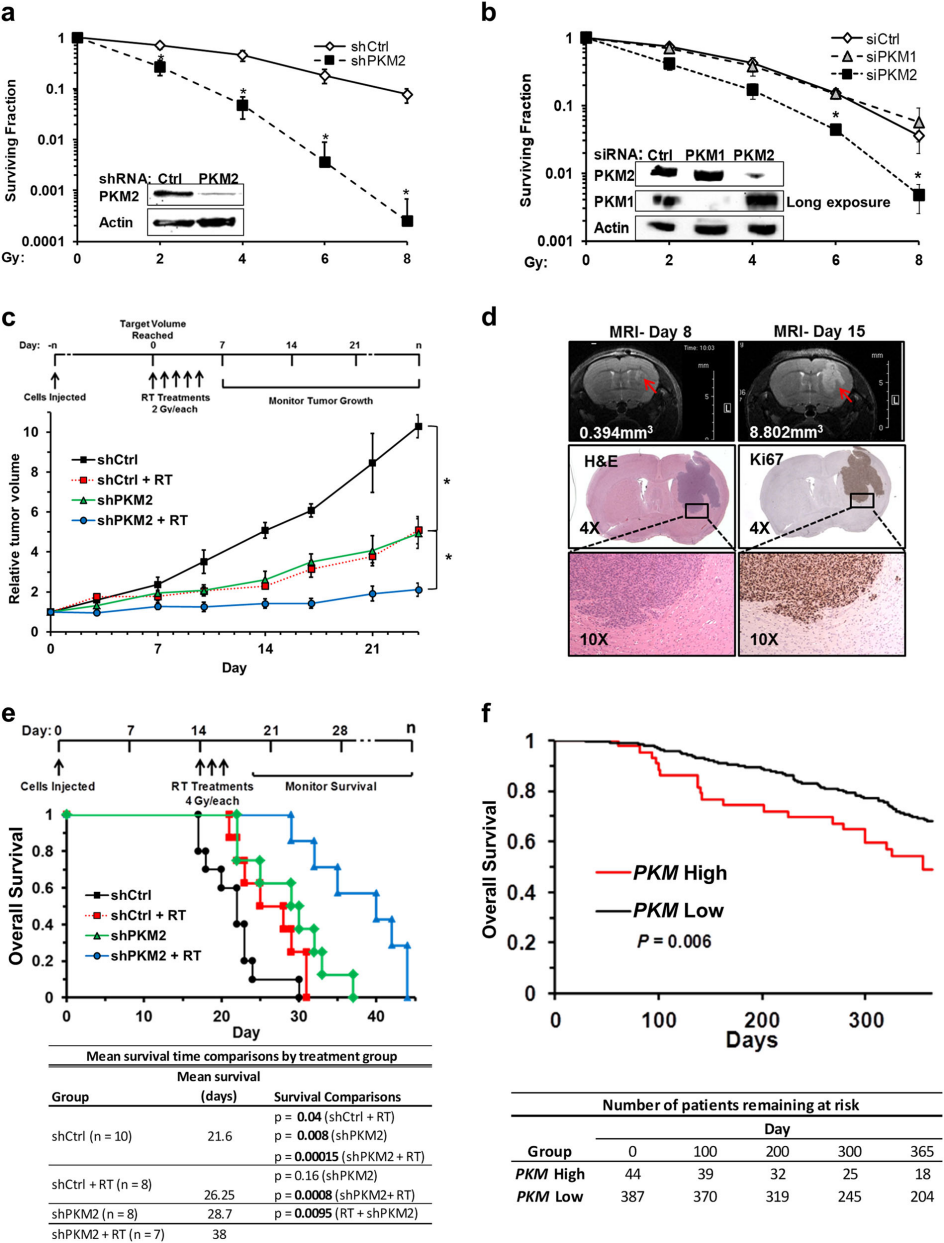
PKM2 is a critical mediator of cellular resistance to DNA-damaging treatment. **a** Clonogenic survival of U87 shCtrl and shPKM2 cells following irradiation with 0 to 8 Gy. Western blot (inset) shows PKM2 expression in these cells. Results are mean ± SE (*n* = 3). *P < 0.05. **b** Clonogenic survival of U87 cells treated with control siRNA or siRNA targeting PKM1 or PKM2 24 h prior to irradiation. Western blot (inset) demonstrates PKM1 and PKM2 expression 24 h after siRNA treatment. Cells were irradiated as indicated. Results are mean ± SE (*n* = 4). *P < 0.05. **c** Mice bearing U87 tumors expressing shCtrl or shPKM2 were assigned to no radiation or radiation treatment (2 Gy × 5 d) groups. The average volume for shCtrl tumors was 132.5 mm^3^ at the start of treatment; for shPKM2 tumors, the average volume at the start of treatment was 137.7 mm^3^. shCtrl tumor reached target volume to begin treatment in 36.5 days on average while shPKM2 tumors required 44.8 days to reach the target volume. Tumor growth was monitored for 24 d. Results are mean tumor volume relative to starting volume ± SD (6 mice/group). *P < 0.05. **d** MRI images of a representative intracranial U87/EGFRvIII tumor at day 8 (upper left panel) and 15 (upper right panel). H&E (middle and lower left panels) and Ki-67 (middle and lower right panels) staining of a day 15 tumor. **e** Mice were implanted with 50,000 U87/EGFRvIII cells expressing shCtrl or shPKM2 by intracranial injection and assigned to no radiation or radiation treatment (4 Gy × 3 d) groups (see Table for details). Mean survival for each group was calculated and survival was analyzed by the Kaplan Meier method, with comparisons between the groups made by a log rank test (see Table). *P* < 0.05 was considered significant. **f** Patients receiving genotoxic treatment in the TCGA GBM cohort were stratified by *PKM* expression (*PKM* High = upper 10th percentile; *PKM* Low = lower 90th percentile) and overall survival was analyzed by the Kaplan Meier method (*P* = 0.006 for the difference between high and low expression groups)

To investigate the role of PKM2 in cancer cell resistance to DNA-damaging therapies, we stably knocked down PKM2 in the human glioblastoma multiforme (GBM) U87 cell line, which expresses high endogenous levels of PKM2^9^ (Supplementary information, Fig. S1a), and subjected these cells to ionizing radiation (IR). While PKM2 knockdown had no effect on cell cycle distribution (data not shown), it resulted in a > 10-fold increase in radiation sensitivity at doses of 4 Gy or above (*P* < 0.005 at 8 Gy, Fig. 1a). Increased sensitivity to radiation following PKM2 knockdown was confirmed by transient knockdown of PKM2 in U87, T98G, and U251 GBM cell lines (Fig. 1b, Supplementary information, Fig. S1b and c), as well as in H1299 (non-small cell lung cancer; data not shown) and HT1904 (derived from the HT1080 fibrosarcoma line; data not shown). In contrast, knockdown of PKM1, the primary pyruvate kinase isoform expressed in normal brain (Supplementary information, Fig. S1a, e, f), did not sensitize U87 cells to radiation (Fig. 1b) nor did PKM1 knockdown sensitize normal human astrocytes to radiation (data not shown).

Next, we examined the role of PKM2 in radiation resistance in vivo by monitoring the growth of subcutaneous U87 tumors expressing shCtrl or shPKM2 in nude mice. The combination of PKM2 knockdown and radiation (2 Gy × 5 d) inhibited tumor growth significantly more than either treatment alone, indicating a critical role for PKM2 in treatment resistance in vivo (Fig. 1c).

We then assessed the role of PKM2 in treatment resistance using a more clinically relevant intracranial tumor model generated with the human GBM U87/EGFRvIII cell line, which expresses a truncated, constitutively active EGFR mutant frequently expressed in GBM (Fig. 1d, e; Supplementary information, Fig. S1d, f). Like actual human GBM tumors (Supplementary information, Fig. S1e), the mouse intracranial U87/EGFRvIII tumor model exhibits rapid proliferation as evidenced by Ki-67 staining (Fig. 1d) and expresses high levels of PKM2 and little PKM1 while, conversely, the surrounding normal brain tissue expresses high levels of PKM1 and little PKM2 (Supplementary information, Fig. S1f). Knockdown of PKM2 alone or whole brain irradiation (4 Gy × 3 d) alone significantly improved mean survival time by 7.1 and 4.65 days, respectively, relative to untreated control mice (Fig. 1e). Importantly, silencing PKM2 in combination with radiation treatment increased the survival time to more than double (16.4 additional days compared to untreated control mice) that attributed to silencing PKM2 alone (7.1 additional days) or radiation alone (4.65 additional days) (Fig. 1e). Combined, these results suggest that PKM2 significantly contributes to the resistance of cancer cells to DNA-damaging treatment in vitro and in vivo.

Radiation with or without temozolomide is standard adjuvant treatment for GBM.^10^ We hypothesized that, as an important mechanism of resistance to DNA-damaging therapies, elevated *PKM2* expression should be associated with decreased overall survival in GBM patients. To test this hypothesis, we selected patients in the large TCGA GBM cohort (TCGA Research Network: http://cancergenome.nih.gov/) that received radiation treatment as well as patients that received no treatment and stratified these populations by *PKM* expression. The probe used in this dataset recognizes the *PKM* transcript which is preferentially spliced in GBM to yield the *PKM2* isoform.^11^ High (upper 10th percentile) *PKM* expression was significantly prognostic of reduced overall survival in patients that received genotoxic treatment (log rank *P* = 0.006; Fig. 1f) but not in those patients that did not receive genotoxic treatment (log rank *P* = 0.09; Supplementary information, Fig. S1g), suggesting an important clinical role of PKM2 in genotoxic treatment resistance.

### PKM2 regulates HR-, but not NHEJ-mediated repair of DSBs

**Fig. 2:**
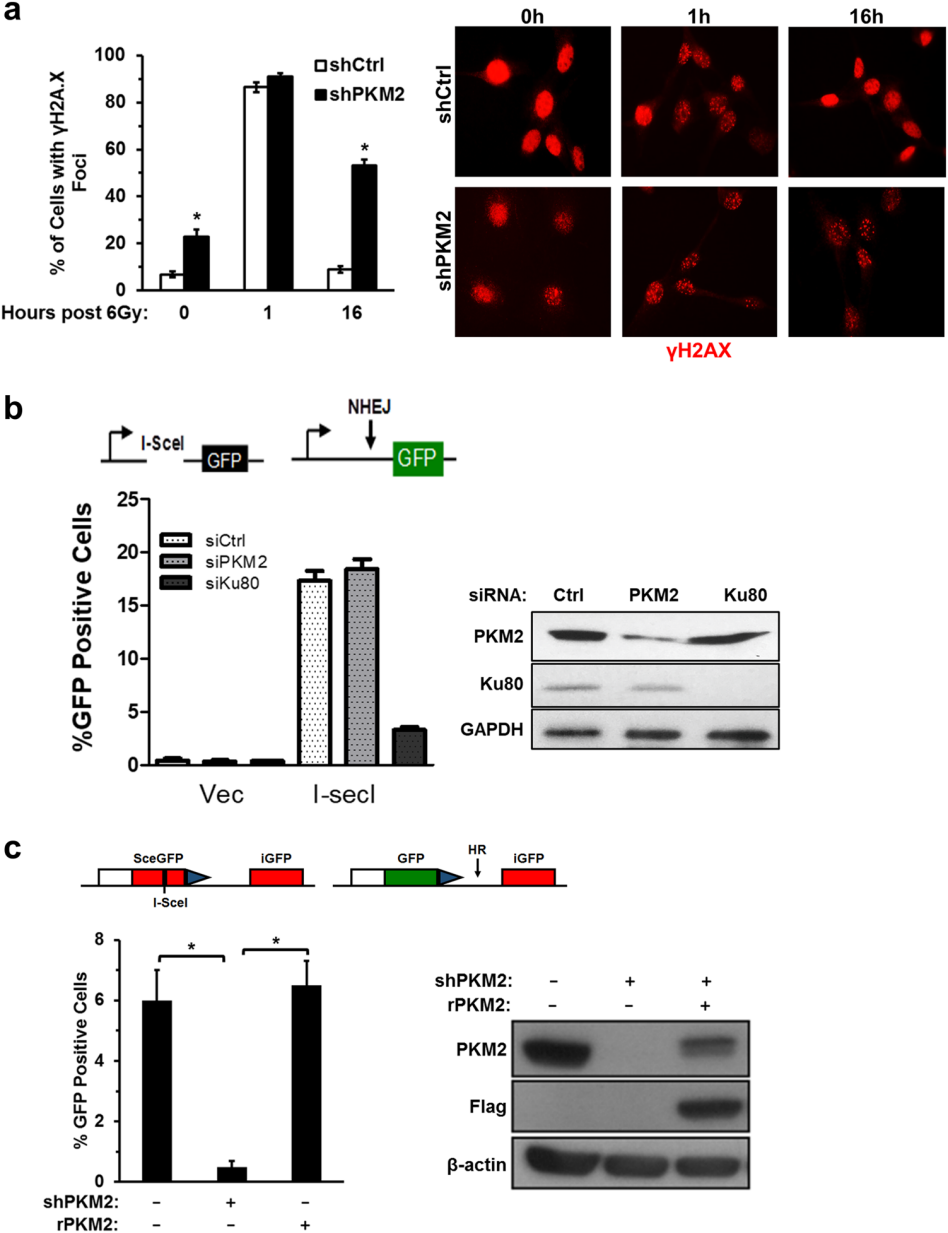
PKM2 regulates HR-, but not NHEJ-mediated repair of DSBs. **a** U87 cells expressing shPKM2 were treated with 6 Gy, fixed, and analyzed for γH2A.X foci at the indicated time points. Data are the mean percentage of cells with foci ± SE (*n* = 3). Representative photos illustrating γH2A.X nuclear foci (red) in shCtrl and shPKM2 cells at 0, 1 and 16 h post treatment with 6 Gy. **b** Repair of DSBs by NHEJ was assayed using the U87 cell line-based chromosomal reporter system containing a single integrated GFP reporter with two I-SceI target sites in inverted orientation flanking an out of frame ATG upstream of the GFP reporter (see schematic). Cleavage by I-SceI removes the out of frame ATG, leaving non-compatible ends. Repair of the DSB by NHEJ results in GFP translation. Cells were transfected with control siRNA or siRNAs targeting PKM2 or KU80 and then transfected with pcDNA3.1/I-SceI. Resolution of DSBs by the NHEJ pathway was assessed by measuring the percentage of GFP-positive cells, with knockdown of KU80 serving as a positive control for disruption of NHEJ repair. Data are mean ± SE (*n* = 3). **c** HR-specific repair of DSBs was analyzed in U87 cells modified with a stably integrated DR-GFP chromosomal reporter system (see schematic). This system contains two differently mutated GFP genes oriented as direct repeats. The upstream repeat contains a single I-SceI site while the downstream copy is a 5′-3′ truncated GFP fragment. Expression of I-SceI in these cells generates a DSB that, when repaired by HR, results in expression of GFP. Cells were treated with control or PKM2-targeting shRNA and transfected with empty vector or vector encoding shRNA-resistant PKM2 (rPKM2). After 24 h, DSBs were induced by transduction with I-SceI-encoding adenovirus and resolution of I-SceI-induced DSBs by the HR pathway was assessed by measuring the percentage of GFP-positive cells 48 h later. Data are presented as mean ± SE (*n* = 3). *P < 0.05

DSBs are the primary source of radiation cytotoxicity. We next asked if PKM2 promoted treatment resistance by promoting the repair of radiation-induced DSBs. Knockdown of PKM2 in U87 cells resulted in an elevated basal level of DSBs, as assessed by γ-H2AX foci (Fig. 2a), suggesting that PKM2 may be involved in managing DSB repair resulting from oxidative stress in the absence of radiation.^12–16^ Interestingly, knockdown of PKM2 did not affect DSBs present 1 h post-irradiation, but did significantly increase the percentage of cells with persistent DSBs remaining 16 h post-treatment (Fig. 2a). This indicates that PKM2 confers treatment resistance not by decreasing the amount of DNA damage incurred as a result of treatment, but rather by encouraging repair of these lesions after the fact.

Repair of DSBs is primarily carried out by either the NHEJ or HR-mediated DNA repair pathways. We utilized chromosomal DSB repair reporter assays, which specifically measure the efficiency of NHEJ repair or HR repair of I-SceI-induced DSBs, respectively, to examine whether PKM2 regulated either of these pathways.^17,18^ Silencing PKM2 had no effect on the efficiency of NHEJ-mediated DSB repair, whereas knockdown of KU80, a positive control, disrupted NHEJ repair in this assay (Fig. 2b). Intriguingly, knockdown of PKM2 significantly diminished HR DSB repair efficiency (Fig. 2c). Given the high level of homology between PKM1 and PKM2 and therefore limited selection of shRNAs specific for either isoform, rescue of PKM2 knockdown by a shRNA resistant PKM2 construct (rPKM2) provided the most definitive method to exclude off-target effects from shPKM2. rPKM2 completely rescued HR DSB repair efficiency in shPKM2 cells (Fig. 2c), demonstrating an essential role for PKM2 in regulating this repair pathway (Fig. 2c).

### PKM2 phosphorylates and activates CtIP to promote HR-mediated DSB repair

**Fig. 3:**
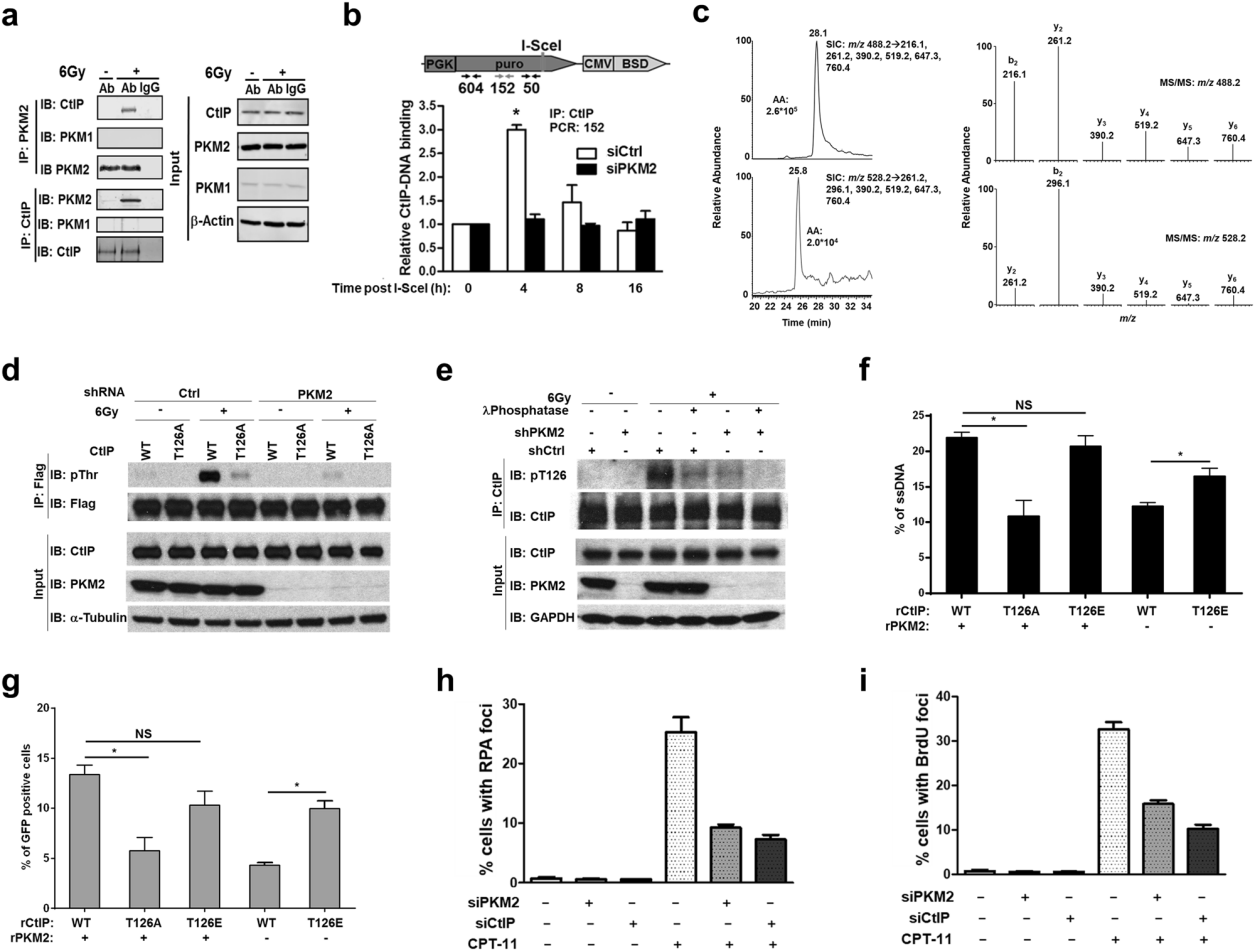
PKM2 phosphorylates and activates CtIP to promote HR-mediated DSB repair. **a** Protein lysates from unirradiated or irradiated U87 cells were collected and immunoprecipitated with PKM2 and CtIP antibodies or anti-rabbit IgG. Immunoprecipitates and input control lysates were analyzed by western blot. **b** Schematic: The I-SceI system in HT1904 cells utilized to analyze CtIP binding upstream of DSBs. ChIP primer sets 604, 152, and 50 bp from the I-SceI site were used to analyze CtIP binding. Bar chart: Cells were transfected with control or PKM2-targeting siRNA and then I-SceI-expressing adenovirus was introduced to create DSBs. At the indicated time points, CtIP-DNA complexes were harvested and CtIP binding sites were quantified by real-time PCR. Data show binding of CtIP at a site 152 bp from the I-SceI-induced DSB normalized to T = 0 and are presented as mean ± SE (*n* = 3). **c** LC-MS/MS in the multiple-reaction monitoring (MRM) mode for monitoring unmodified CtIP peptide, NTLQEENK, and the same peptide with Thr^126^ being phosphorylated in the irradiated sample. Selected-ion chromatograms for monitoring the indicated transitions arising from the fragmentations of the [M + 2 H]^2+^ ions of the unphosphorylated (top left) and phosphorylated (bottom left) NTLQEENK. MS/MS for the [M + 2 H]^2+^ ions of the unphosphorylated (top right) and phosphorylated (bottom right) peptides averaged from the peaks at around 28.1 min and 25.8 min, respectively. **d** Protein lysates from U87 Ctrl and shPKM2 cells expressing flag-tagged WT or T126A CtIP and treated with 0 or 6 Gy radiation were immunoprecipitated with anti-flag antibody and separated by SDS-PAGE followed by immunoblotting with flag and pan-phosphothreonine antibodies. Input protein lysates were collected prior to immunoprecipitation. **e** Protein lysates from U87 cells treated with 0 or 6 Gy radiation were immunoprecipitated with anti-CtIP antibody and separated by SDS-PAGE followed by immunoblotting with pT126 CtIP and total CtIP antibodies. Input protein lysates were collected prior to immunoprecipitation. Lysates from U87 cells treated with 6 Gy were also pre-treated with λ-phosphatase prior to immunoprecipitation with anti-CtIP antibody to demonstrate specificity of the pT126 CtIP antibody for the phosphorylated protein. Input protein lysates were collected prior to immunoprecipitation. **f** HT1904 cells containing the I-SceI DSB system were utilized to analyze DNA end-resection adjacent to I-SceI-induced DSBs. Endogenous CtIP and PKM2 were knocked down and replaced with WT, T126A, or T126E CtIP and PKM2 as indicated. After 24 h, DSBs were induced by adenoviral delivery of I-SceI. Genomic DNA was isolated 4 h later and ssDNA adjacent to I-SceI-induced DSBs was quantitated by quantitative real-time PCR. Data are presented as mean ± SE (*n* = 3). **g** Endogenous CtIP and PKM2 were knocked down by siRNA or shRNA, respectively, in U87 DR-GFP cells and replaced by WT, T126A, or T126E CtIP and PKM2 as indicated. After 24 h, DSBs were induced by adenoviral delivery of I-SceI and the ability of cells to resolve DSBs by the HR pathway was assessed by measuring the percentage of GFP-positive cells 48 h later. Data are presented as mean ± SE (*n* = 3). *P < 0.05. NS, not significant. **h** PKM2 or CtIP was knocked down in U87 cells by siRNA. Cells were then treated with DMSO or 2 µM CPT-11 for 1 h then stained with antibodies against phosphoS4/S8-RPA32. Cells with > 10 foci were considered positive. **i** PKM2 or CtIP were knocked down in U87 cells by siRNA. Cells were treated with BrdU (10 mg/mL) for 16 h and then with DMSO or CPT-11 (2 µM) for 1 h prior to fixation. Cells with > 10 foci were considered positive

To illuminate how PKM2 promotes HR-mediated DSB repair, we examined if PKM2 interacted with well-known mediators of HR repair. A number of HR repair proteins, including Brca1, Mre11, Rad51, RPA, and CtIP, co-immunoprecipitated with PKM2 (Fig. 3a; Supplementary information, Fig. S2a and b) in a DNA-damage dependent manner. Given the essential role of CtIP in HR repair of DSBs,^19^ we focused our analyses on PKM2’s ability to regulate CtIP function during HR repair. First, we assessed CtIP binding to DNA 50–604 nucleotides upstream of I-SceI-induced DSBs^20^ (Fig. 3b). CtIP binding to DNA flanking DSBs was increased 3-fold at 4 h after adenoviral delivery of I-SceI and returned to basal levels by 8 h (Fig. 3b; Supplementary information, Fig. S2c). PKM2 knockdown significantly impaired CtIP recruitment to DSBs, indicating that PKM2 regulates this important function of CtIP.

In addition to catalyzing the transfer of a phosphate group from phosphoenolpyruvate (PEP) to ADP to produce pyruvate and ATP in the cytoplasm, PKM2 functions as a nuclear protein kinase utilizing PEP as a phosphate donor.^21^ Given that phosphorylation is an important mechanism for regulating CtIP’s function,^19^ we analyzed if PKM2 phosphorylated CtIP. We observed a PKM2-dependent increase in threonine, but not tyrosine or serine phosphorylation of CtIP in irradiated U87 cells (Supplementary information, Fig. S2d and data not shown). To identify the threonine residue(s) of CtIP that are phosphorylated by PKM2, we analyzed phosphorylation of recombinant protein fragments covering the N-terminal (residues 45–371), middle (residues 374–649) and C-terminal (residues 650–897) portions of CtIP in the presence of recombinant PKM2 in vitro (Supplementary information, Fig. S2e). WT and R399E PKM2, which has increased protein kinase activity,^6^ utilized PEP, but not ATP, as a phosphate donor to phosphorylate the N-terminal region of CtIP in vitro, while the mid-portion and the C-terminal part were not phosphorylated (Supplementary information, Fig. S2e). We next used a series of additional CtIP peptide fragments to narrow the threonine residue on CtIP phosphorylated by PKM2 to the region between residues 121 and 180 (Supplementary information, Fig. S2f). Two threonine residues (T126 and T166) are located in this region. We mutated each of these residues in fragment 61–274 to an alanine and found that only T126 was phosphorylated by PKM2 (Supplementary information, Fig. S2g and data not shown). To confirm that CtIP was phosphorylated in a PKM2-dependent manner on T126 following radiation, we conducted liquid chromatography-coupled ion mass spectrometry (LC-MS/MS) analysis of trypsin-digested fragments of Flag-CtIP overexpressed in U87 control or shPKM2 cells with or without radiation treatment (Fig. 3c; Supplementary information, Fig. S3a and b). LC-MS/MS in the multiple-reaction monitoring (MRM) mode revealed that phosphorylation of the CtIP T126-containing peptide, NTLQEENK, was stimulated by exposure to radiation and this stimulation was abrogated in cells after shRNA-mediated knockdown of PKM2. To further evaluate T126 as the specific residue of CtIP phosphorylated by PKM2 following DNA damage, we expressed flag-tagged WT or T126A CtIP in shPKM2 U87 cells. Radiation resulted in a significant increase in threonine phosphorylation of WT but not T126A CtIP and this phosphorylation was abrogated by knockdown of PKM2 (Fig. 3d), further confirming T126 as the unique residue on CtIP phosphorylated by PKM2 in response to DNA damage. Finally, to analyze PKM2-mediated phosphorylation of T126 on endogenous CtIP in response to radiation we utilized an antibody specific for phosphorylated T126 CtIP (Fig. 3e). A significant increase in endogenous pT126 CtIP was observed in protein lysates harvested from cells following radiation treatment (Fig. 3e) or treatment with the topoisomerase I inhibitor CPT-11 (irinotecan) (Supplementary information, Fig. S4a). Treating these lysates with λ-phosphatase significantly reduced the intensity of the band detected by anti-pT126 CtIP antibody following immunoblotting suggesting that this antibody is specific for the phosphorylated protein. Furthermore, knockdown of PKM2 significantly reduced the amount of endogenous pT126 CtIP detected in the lysates of irradiated cells (Fig. 3e), whereas knockdown of PKM1 did not reduce T126-CtIP phosphorylation following DNA damage (Supplementary information, Fig. S4a).

We next asked if phosphorylation of T126 by PKM2 regulated CtIP function in HR repair. To begin, we used an in vivo chromosome-based ssDNA resection assay to analyze CtIP-mediated resection at I-SceI-generated DSBs (Supplementary information, Fig. S4b). In this assay the relative amount of ssDNA adjacent to I-SceI sites, indicative of CtIP end-resection activity, is measured by quantitative PCR.^22^ Endogenous PKM2 and CtIP were knocked down and replaced as indicated by flag-tagged versions of WT PKM2 (rPKM2) and either WT CtIP, T126A CtIP, or phosphomimetic T126E CtIP (Fig. 3f). In cells expressing rPKM2, T126E CtIP fully restored ssDNA resection function relative to cells expressing WT CtIP, whereas expression of an equivalent amount of T126A CtIP resulted in significantly reduced ssDNA resection (Fig. 3f, columns 1–3). Importantly, in cells lacking PKM2, expression of phosphomimetic T126E resulted in significantly greater resection function compared to expression of an equivalent amount of WT CtIP, providing evidence that phosphorylation of CtIP at T126 by PKM2 increases CtIP resection function (Fig. 3f, columns 4–5). We next analyzed if phosphorylation of CtIP T126 by PKM2 regulated HR-mediated repair of I-SceI-induced DSBs (Fig. 3g). Similar to the results of the DNA resection assay, in the presence of PKM2, expression of T126E restored HR repair function to a level similar to cells expressing WT CtIP, while expression of an equivalent amount of T126A CtIP resulted in significantly reduced HR-mediated DSB repair (Fig. 3g, columns 1–3). Furthermore, expression of T126E CtIP in cells lacking PKM2 resulted in significantly greater HR-mediated DSB repair compared to expression of WT CtIP (Fig. 3g, columns 4–5). These results establish phosphorylation of CtIP at T126 by PKM2 as a critical mechanism to activate CtIP’s function in HR-mediated DSB repair. To further confirm a role for PKM2 in the regulation of CtIP function, we analyzed CtIP-regulated DNA end resection by fluorescence microscopy to visualize ssDNA-bound RPA foci and detection of BrdU-ssDNA tracks following exposure to irinotecan (CPT-11) (Fig. 3h, i; Supplementary information, Fig. S4c and d). Knockdown of PKM2 significantly reduced DNA end resection as determined by these methods.

### ATM phosphorylates nuclear PKM2 leading to its retention in the nucleus

**Fig. 4:**
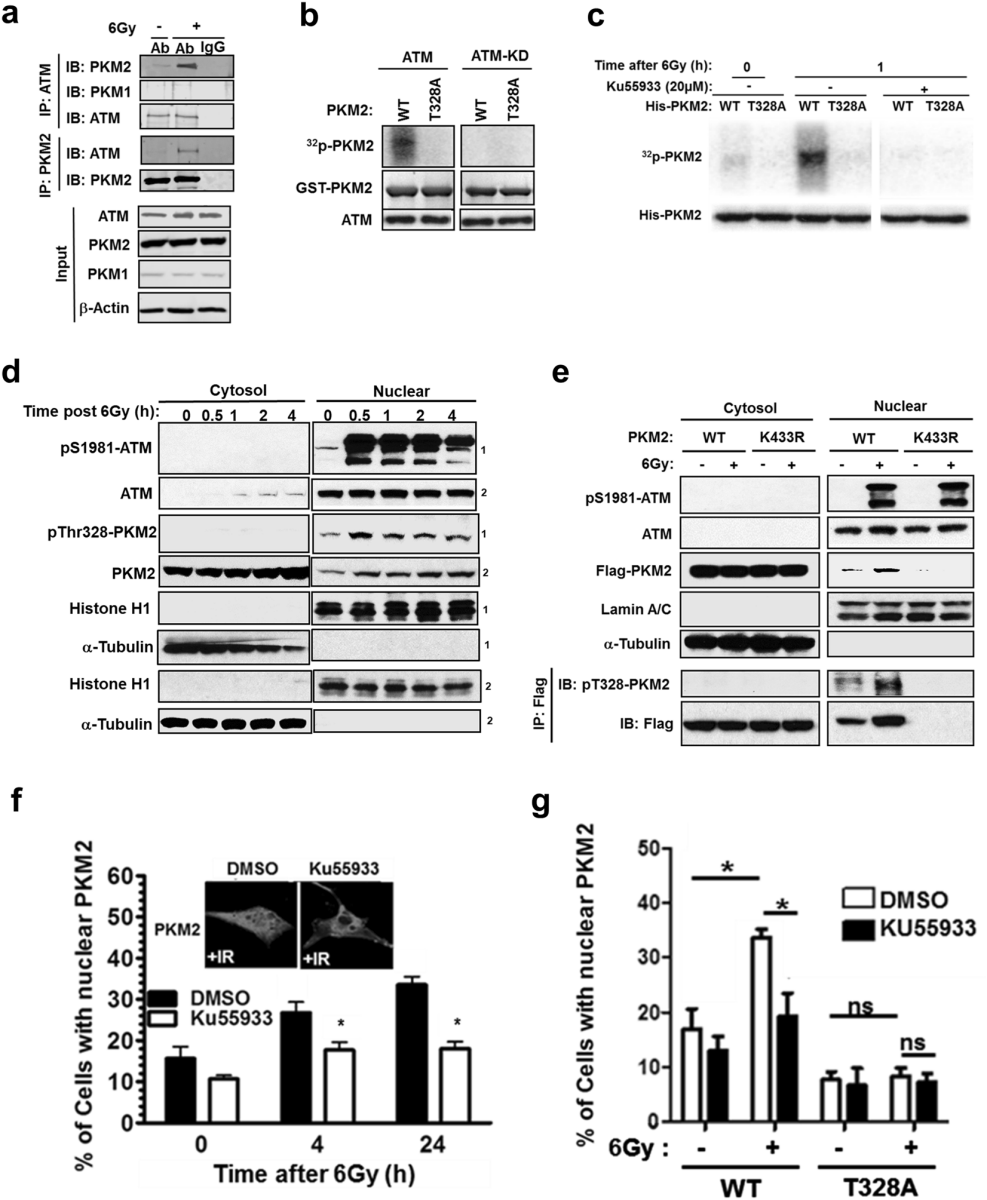
ATM phosphorylates nuclear PKM2 leading to its retention in the nucleus. **a** Protein lysates from U87 cells treated with 0 or 6 Gy were immunoprecipitated with antibodies against ATM, PKM2, or anti-rabbit IgG. Immunoprecipitates and protein input control lysates were separated by SDS-PAGE and then immunoblotted with the indicated antibodies. **b** Recombinant GST-tagged WT or T328A PKM2 were incubated with isolated ATM or a kinase-dead ATM (ATM-KD) in the presence of [γ-^32^P]ATP. Proteins were separated by SDS-PAGE, and ^32^P-radiolabeled PKM2 was visualized using a PhosphorImager while PKM2 and ATM were detected by immunobloting. **c** His-tagged WT or T328A PKM2 was expressed in 293 T cells incubated in media containing [γ-^32^P]ATP for 4 h prior to exposure to 6 Gy. KU55933 (20 µM) was introduced 1 h prior to radiation. Lysates were collected 1 h after irradiation and resolved by SDS-PAGE, and radiolabeled proteins were visualized by PhosphorImager. His-tagged PKM2 was detected by immunoblotting. **d** U87 cells were treated with 6 Gy radiation and cell lysates were collected at the indicated time points and separated into nuclear and cytoplasmic fractions, which were resolved by SDS-PAGE and probed with the indicated antibodies. Superscripts 1 and 2 identify respective membranes used for immunoblotting. **e** U87 cells expressing flag-tagged WT or K433R PKM2 were treated with 0 or 6 Gy radiation. Cell lysates were collected 45 min after treatment and separated into nuclear and cytoplasmic fractions, which were resolved by SDS-PAGE and probed with antibodies as indicated. Cytosolic and nuclear fractions were also subjected to co-immunoprecipitation with anti-flag antibody prior to being resolved by SDS-PAGE and probed with antibodies against pT328-PKM2 and Flag. **f** U87 cells were treated with DMSO or KU55933 (20 µM) prior to irradiation. At the indicated time points, cells were fixed and PKM2 localization was determined by immunohistochemical staining. Photographs represent typical nuclear localization of PKM2 seen following exposure to radiation, which is prevented by KU55933. Data are presented as the mean percentage of cells with nuclear PKM2 staining ± SE (*n* = 3). **g** U87 shPKM2 cells were transfected with shRNA-resistant WT or T328A PKM2, and then treated with DMSO or KU55933 (20 µM) prior to irradiation as indicated. Cells were fixed and PKM2 localization was determined by immunohistochemical staining. Data are presented as the mean percentage of cells with nuclear PKM2 staining ± SE (*n* = 3). *P < 0.05. ns, not significant

Next, we inquired how the presence of DNA damage is signaled to PKM2 to activate its role in DSB repair. ATM is a well-established critical early response kinase activated in response to DNA damage^3^ and T328 of PKM2 was previously identified as a potential ATM phosphorylation site.^23^ We observed, via reciprocal co-immunoprecipitation experiments, that IR induces ATM binding to PKM2, but not PKM1 (Fig. 4a). Furthermore, purified ATM phosphorylated WT PKM2 but not T328A PKM2, while the kinase-dead version of ATM (ATM-KD) did not phosphorylate either WT or T328A PKM2, confirming T328 as a target residue for phosphorylation by ATM (Fig. 4b). To determine if ATM phosphorylates T328 on PKM2 following DNA damage in vivo, we expressed His-tagged WT or T328A PKM2 in 293 T cells grown in the presence of radiolabeled ATP. Radiation induced a significant increase in the phosphorylation of WT but not T328A PKM2, which was prevented by the ATM inhibitor KU55933 (Fig. 4c). Combined, these data reveal that, in response to DNA damage, ATM phosphorylates PKM2 on T328.

Both ATM and PKM2 shuttle between the cytoplasmic and nuclear compartments in response to a variety of stimuli.^5,24^ While the majority of PKM2 is cytoplasmic, ATM is predominantly nuclear.^25^ We next investigated where DNA damage-induced ATM-mediated phosphorylation of PKM2 occurred. Western blot analysis of cytosolic and nuclear protein fractions revealed that phosphorylation on T328 can be detected by an antibody specific for this phosphorylated residue (Supplementary information, Fig. S5a) as soon as 30 min following DNA damage generated by either radiation (Fig. 4d) or hydrogen peroxide (Supplementary information, Fig. S5b). Phosphorylated T328 PKM2 persists in the nucleus for up to 24 h after induction of DNA damage (Supplementary information, Fig. S5c). Consistent with previous reports,^26–28^ we observed an increase in S1981-phosphorylated ATM levels in the nucleus following radiation and hydrogen peroxide treatment (Fig. 4d; Supplementary information, Fig. S5b). Importantly, pT328 PKM2 is only detected in the nucleus, suggesting that phosphorylation of PKM2 by ATM is a nuclear event. To confirm that phosphorylation of PKM2 by ATM occurred in the nucleus, we analyzed the phosphorylation of flag-tagged WT PKM2 or a nuclear translocation-deficient mutant PKM2 (K433R).^6^ While phosphorylation of WT PKM2 at T328 was observed in nuclear protein fractions and increased in response to radiation, K433R PKM2 was confined to the cytoplasm and T328 phosphorylation of K433R PKM2 even following radiation was not detected (Fig. 4e; Supplementary information, Fig. S5d). Combined these data suggest that ATM phosphorylates PKM2 in the nucleus.

Interestingly, we also observed that T328 phosphorylation is associated with PKM2 nuclear accumulation (Fig. 4d, e). Given that PKM2 promotes HR repair of DSBs by phosphorylating CtIP, a primarily nuclear protein,^29^ we hypothesized that ATM-mediated phosphorylation of PKM2 regulated PKM2’s nuclear accumulation. In support of this hypothesis, PKM2 (Supplementary information, Fig. S6a-d), but not PKM1 (Supplementary information, Fig. S6c and d), accumulated in the nucleus following radiation as determined by both immunofluorescence staining and western blot analysis of nuclear and cytoplasmic protein fractions. This accumulation was significantly prevented by the ATM inhibitor KU55933 (Fig. 4f; Supplementary information, Fig. S6d). Furthermore, T328A PKM2 failed to accumulate in the nucleus after radiation, and KU55933 had no effect on its localization (Fig. 4g; Supplementary information, Fig. S6e), illustrating that nuclear accumulation of PKM2 following radiation requires phosphorylation of T328 by ATM. T328A PKM2 accumulated in the nucleus in a similar manner to WT PKM2 following EGF stimulation which has previously been demonstrated to be dependent upon S37 of PKM2^6^ (Supplementary information, Fig. S6f), illustrating that T328A PKM2 was capable of nuclear accumulation in response to stimuli other than DNA damage. Combined, these data support a model in which ATM phosphorylates PKM2 in the nucleus, leading to its nuclear accumulation and increased repair of DNA damage resulting from genotoxic treatments.

### ATM-dependent phosphorylation of PKM2 on T328 regulates PKM2’s capacity to activate of CtIP and HR repair

**Fig. 5:**
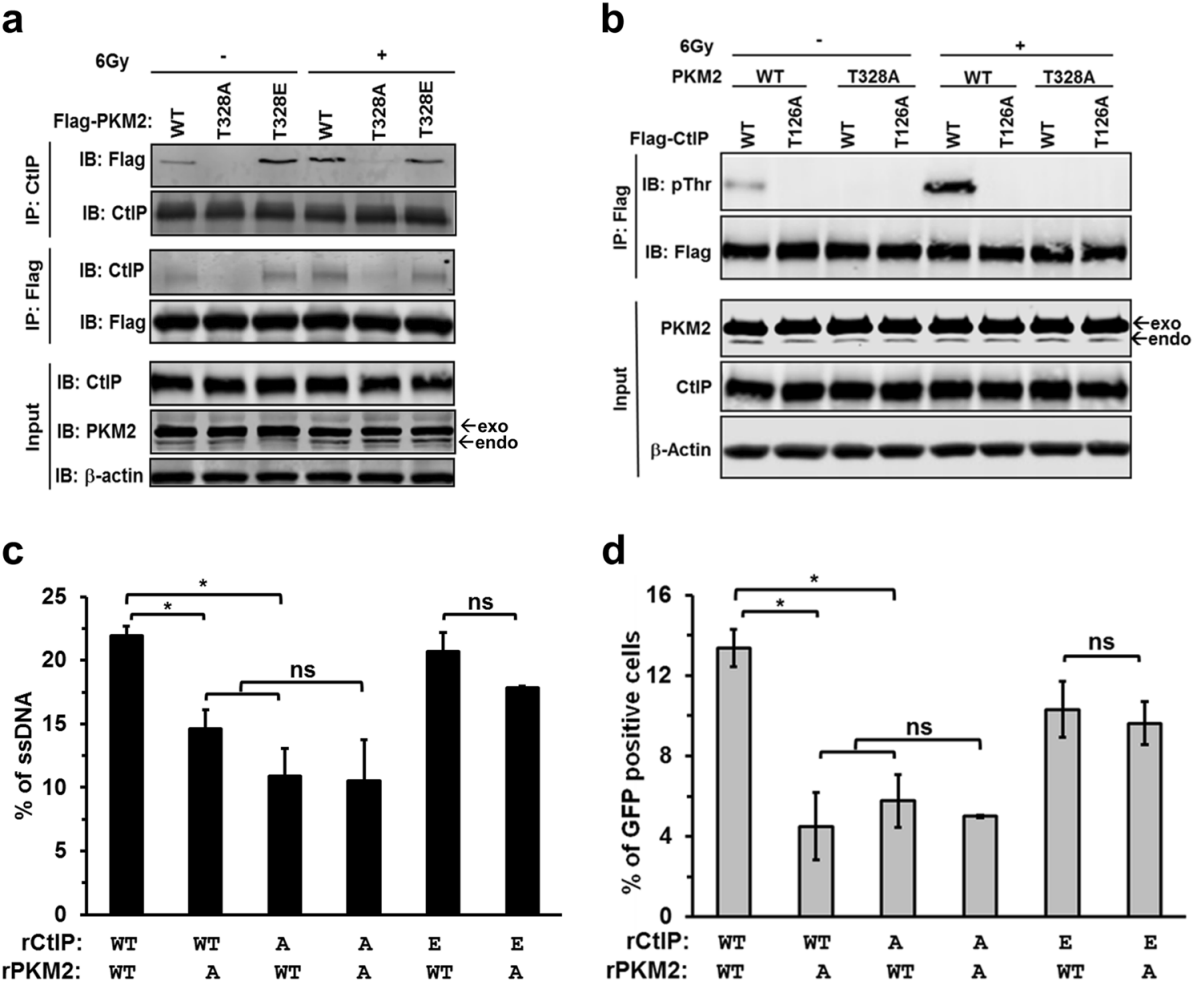
ATM phosphorylation of PKM2 on T328 regulates PKM2’s activation of CtIP and HR repair. **a** shRNA-resistant, flag-tagged WT, T328A or T328E PKM2 were expressed in U87 shPKM2 cells. Cells were irradiated, and cell lysates were collected and immunoprecipitated with antibodies against CtIP or Flag. Immunoprecipitates and input protein lysates were resolved by SDS-PAGE, and then probed with the indicated antibodies. exo/endo, exogenously or endogenously expressed PKM2. **b** shRNA-resistant WT or T328A PKM2 was expressed in U87 shPKM2 cells along with flag-tagged WT or T126A CtIP as indicated. Cells were treated with 0 or 6 Gy, and cell lysates were collected and immunoprecipitated with anti-flag antibody. Immunoprecipitates and input lysates were resolved by SDS-PAGE, then probed with the indicated antibodies. exo/endo, exogenously or endogenously expressed PKM2. **c** HT1904 cells containing the I-SceI DSB system were used to analyze DNA end-resection adjacent to I-SceI-induced DSBs. Endogenous CtIP and PKM2 were knocked down and replaced with WT, T126A (A), or T126E (E) CtIP and WT or T328A (A) PKM2 as indicated. DSBs were induced by adenoviral delivery of I-SceI. Genomic DNA was isolated, and ssDNA adjacent to I-SceI-induced DSBs was quantitated by quantitative real-time PCR. Data are presented as mean ± SE (*n* = 3). **d** Endogenous CtIP and PKM2 were knockdown by siRNA or shRNA in U87 DR-GFP cells and replaced by WT, T126A (A) or T126E CtIP (E) and WT or T328A (A) PKM2. DSBs were induced by adenoviral delivery of I-SceI, and the ability of cells to resolve DSBs by the HR pathway was assessed by the percentage of GFP-positive cells 48 h later. Data are presented as mean ± SE (*n* = 3). *P < 0.05. ns, not significant

We next asked if phosphorylation of T328 by ATM was necessary for the interaction of PKM2 with CtIP. Endogenous PKM2 was replaced in U87 cells by flag-tagged WT, T328A, or T328E PKM2. In the absence of radiation, minimal interaction was detected between WT PKM2 and CtIP, and no binding between T328A PKM2 and CtIP was detected (Fig. 5a). In contrast, significantly more T328E PKM2, compared to WT PKM2, was found in complex with CtIP in the absence of radiation (Fig. 5a). Radiation significantly increased the interaction of WT PKM2 with CtIP, but had no effect on the interaction of either T328A or T328E PKM2 with CtIP (Fig. 5a). In addition, KU55933 reduced the association of PKM2 and CtIP by greater than 2.5-fold after irradiation (Supplementary information, Fig. S7a). Next, we determined if ATM-dependent phosphorylation of PKM2 on T328 is required for PKM2 to phosphorylate CtIP on T126. Endogenous PKM2 and CtIP were knocked down in U87 cells and replaced by flag-tagged WT or T328A PKM2 and WT or T126A CtIP. Irradiation resulted in a significant increase in CtIP phosphorylation that was dependent upon the presence of both T328 on PKM2 and T126 on CtIP (Fig. 5b). Together, these results reveal that phosphorylation of PKM2 on T328 by ATM following DNA damage is required for PKM2 to form a complex with and phosphorylate CtIP on T126.

To better understand the extent to which the ATM-PKM-CtIP axis contributes to the PKM2-mediated increase in DSB repair, we analyzed the resection efficiency of I-SceI-induced DSBs in HT1904 cells in which endogenous PKM2 and CtIP were both knocked down and replaced with WT or T328A PKM2 and WT, T126A, or T126E CtIP. Replacing either WT PKM2 with T328A PKM2 or WT CtIP with T126A CtIP significantly impaired resection (Fig. 5c, columns 2–3 versus column 1; Supplementary information, Fig. S7b). Replacing both WT PKM2 and WT CTIP with T328A PKM2 and T126A CtIP did not further reduce resection efficiency compared to cells in which only one of these proteins was mutated (column 4 versus columns 2–3), suggesting they function in the same pathway. Furthermore, resection function was not significantly different between cells expressing WT or T328A PKM2 when phosphomimetic T126E CtIP was expressed (columns 5–6), demonstrating that phosphorylation of T126 on CtIP by PKM2 is important for CtIP function. Similar results were found in complementary assays measuring HR-mediated repair of I-SceI-induced DSBs (Fig. 5d; Supplementary information, Fig. S7c). Furthermore, KU55933 significantly decreased HR repair in cells expressing WT PKM2, but had no significant effect on HR-mediated repair in cells expressing the T328A or T328E PKM2 mutants (Supplementary information, Fig. S7d). Additionally, cells expressing T328E PKM2 retained a significantly greater portion of their HR repair function following KU55933 treatment than those expressing WT PKM2 (Supplementary information, Fig. S7d). Taken together, these data demonstrate that ATM-mediated phosphorylation of PKM2 at T328 is prerequisite for PKM2 to phosphorylate CtIP on T126 and increase HR repair of DSBs.

### The ATM-PKM2-CtIP axis provides resistance to genotoxic agents

**Fig. 6:**
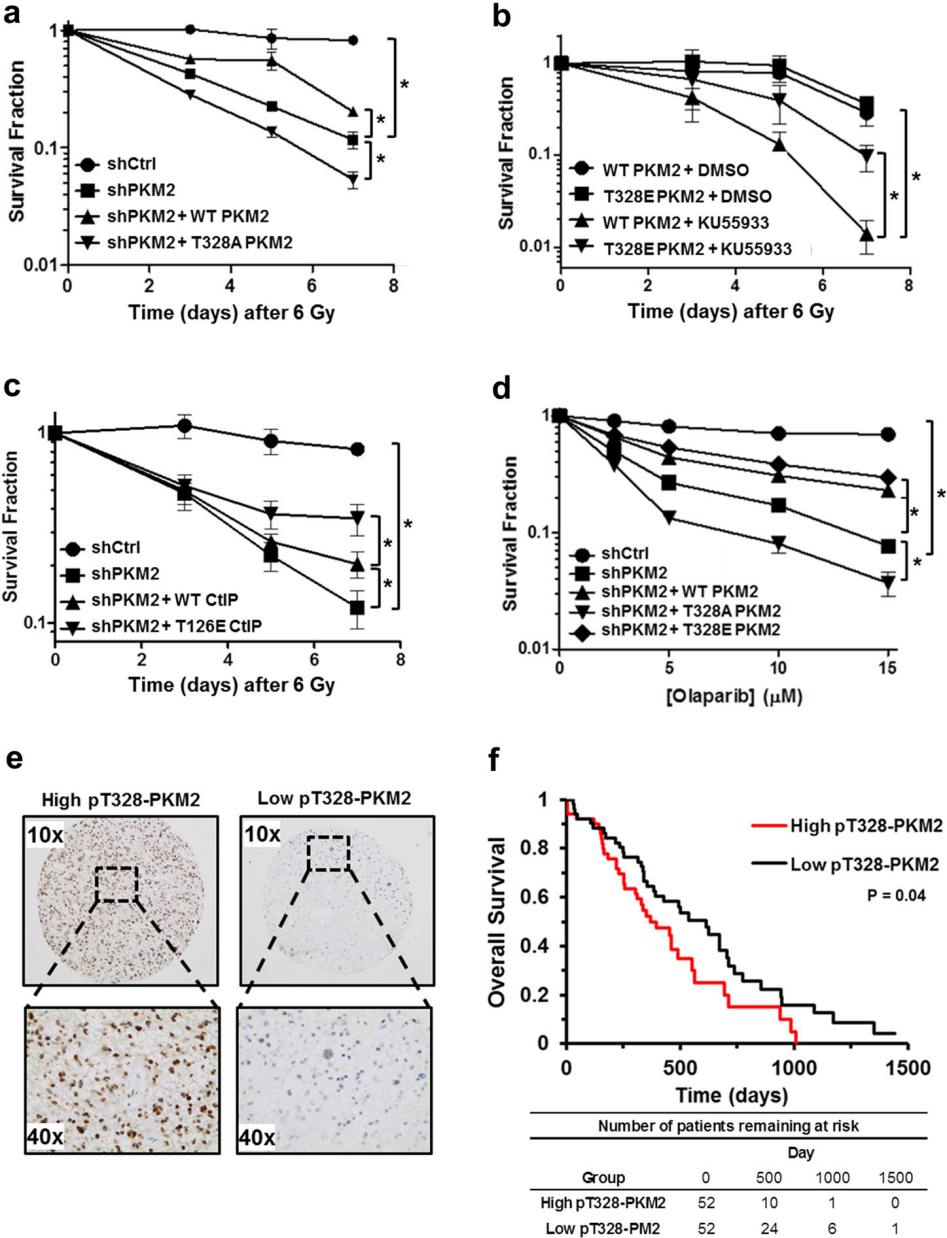
The ATM-PKM2-CtIP axis provides resistance to genotoxic agents. **a** U87 shCtrl and shPKM2 cells, and shPKM2 cells expressing shRNA-resistant WT or T328A PKM2, were irradiated with 6 Gy. The number of viable cells was determined by trypan blue staining at the indicated time points. Data are the relative mean fraction of surviving cells ± SE (*n* = 3). **b** WT or T328E PKM2 was overexpressed in U87 cells. Cells were pre-treated with DMSO or 10 µM KU55933 and then irradiated. The number of viable cells was determined by trypan blue staining at the indicated time points. Data are the relative mean fraction of surviving cells ± SE (*n* = 3). **c** U87 shCtrl or shPKM2 cells, and shPKM2 cells overexpressing WT or T126E CtIP, were irradiated and the number of viable cells was determined by trypan blue staining at the indicated time points. Data are the relative mean fraction of surviving cells ± SE (*n* = 3). **d** U87 shCtrl or shPKM2 cells, and shPKM2 cells expressing shRNA-resistant WT, T328A, or T328E PKM2 were incubated with the indicated concentrations of olaparib. Cell viability was determined by trypan blue staining 48 h after treatment. Data are the relative mean fraction of surviving cells ± SE (*n* = 3). **e** Representative 10 × and 40 × images from GBM patient samples exhibiting high (left panels) or low (right panels) staining for pT328-PKM2. **f** The GBM patient cohort (*n* = 104) was separated into two groups along the median pT328-PKM2 H-score. Survival was analyzed by the Kaplan Meier method. Statistical differences in overall survival between the groups were determined by the log rank test (*P* = 0.04). *P < 0.05

Our results thus far have established an important novel function of PKM2 in regulating HR-mediated DSB repair. We next asked if signaling through the newly discovered ATM-PKM2-CtIP repair axis rendered cancer cells resistance to a variety of genotoxic agents. First, endogenous PKM2 was knocked down in U87 cells and replaced by WT or T328A PKM2 (Supplementary information, Fig. S8a). These cells were subjected to 6 Gy radiation and monitored for survival over 7 days (Fig. 6a). As expected, knockdown of PKM2 significantly decreased survival, which could be partially, but significantly, rescued by expression of exogenous WT PKM2. Conversely, T328A PKM2 expressed at a similar level (Supplementary information, Fig. S8a) not only did not rescue survival in PKM2 knockdown cells but further reduced survival.

Since T328 is the target site for phosphorylation by ATM on PKM2, we next tested if KU55933 sensitized U87 cells to radiation treatment and if this sensitization could be reversed by expression of phosphomimetic T328E PKM2. As expected, KU55933 sensitized U87 cells expressing WT PKM2 to 6 Gy radiation (Fig. 6b). Replacing WT PKM2 with T328E PKM2 expressed at a similar level (Supplementary information, Fig. S8a) significantly increased cell survival after treatment (Fig. 6b). Given that PKM2 regulates HR repair of DSBs by activating CtIP through phosphorylation of T126, we next asked if expression of phosphomimetic T126E CtIP could restore treatment resistance in PKM2 knockdown cells. While overexpression of WT CtIP conferred some resistance to radiation treatment in PKM2 knockdown cells, expression of an equivalent amount of T126E CtIP (Supplementary information, Fig. S8b) provided significantly greater resistance (Fig. 6c). Collectively, these results demonstrate that the ATM-PKM2(T328)-CtIP(T126) axis is a significant contributor to cancer cell resistance to DNA-damaging therapies.

HR-mediated DSB repair deficiency in cancer cells can create a synthetic lethality to PARP inhibitors (PARPi). We found that knockdown of PKM2 increased sensitivity of U87 (Fig. 6d) and U251 (Supplementary information, Fig. S8c) to the PARPi olaparib. Overexpression of either WT or T328E PKM2 rescued U87 and U251 shPKM2 cells from PARPi synthetic lethality, whereas T382A expression actually increased the sensitivity of PKM2 knockdown cells to PARPi. Additional experiments in which U87 cells were subjected to a range of radiation doses from 0 to 6 Gy (Supplementary information, Fig. S8d), treated with the topoisomerase I inhibitor CPT-11 (irinotecan) to induce DNA damage (Supplementary information, Fig. S8e and f), or with hydrogen peroxide (Supplementary information, Fig. S8g) to produce oxidative stress confirmed that exogenous WT PKM2 significantly rescued survival in PKM2 knockdown cells while equivalent expression of T328A PKM2 further impaired survival.

Finally, we asked if ATM-dependent phosphorylation of PKM2 at T328 was prognostic of GBM patient clinical outcome. We stained a cohort of GBM tumors with antibodies against pS1981-ATM and pT328-PKM2 (Supplementary information, Fig. S9a). Interestingly, even though these samples were taken from patients prior to therapy, we found a significant correlation (*P* = 0.005) between pS1981-ATM and nuclear pT328-PKM2 staining (Supplementary information, Fig. S9b), which may reflect an important role for this pathway in coping with increased oxidative stress within the tumor environment. Importantly, elevated nuclear pT328-PKM2 was significantly prognostic of worse overall survival (log rank *P* = 0.04; Fig. 6e, f) suggesting that activation of this pathway might identify tumors that are primed for resistance to genotoxic therapies.

## Discussion

Glucose metabolism and DNA repair are two fundamental cellular processes frequently dysregulated in cancer. Metabolic pathways provide cells with nucleic acids and energy required to repair DNA. As such, it is not surprising that crosstalk exists to coordinate these two essential networks.^8,30^ However, until now, the connections between the metabolic and DNA repair circuitries have been limited. We show that PKM2, the key enzyme of glycolysis, is a direct target for phosphorylation by ATM. Importantly, this phosphorylation event is essential for PKM2’s retention in the nucleus, where PKM2 phosphorylates CtIP, one of the principal components of HR repair, creating a novel direct connection between glycolytic metabolism and DNA repair.

PKM2’s function as a threonine and tyrosine protein kinase has been demonstrated in a number of previous reports. Yang et al. initially demonstrated that phosphorylation of histone H3 at T11 directly by PKM2 facilitates the dissociation of HDAC3 from the promoters of β-catenin target genes.^31^ PKM2 has also been reported to phosphorylate STAT3 at Y705, thereby enhancing its transcriptional activity.^16^ More recently, PKM2 has been found to phosphorylate H2AX,^32^ PAK2,^33^ and SNAP-23.^34^ While the ability of PKM2 to directly phosphorylate proteins has been challenged,^35^ unbiased proteomic screens have identified over 900 substrates for PKM2.^36,37^ Our work establishes PKM2 as a novel upstream regulator of CtIP and its direct phosphorylation of CtIP on T126 as an important mechanism of activation of CtIP-associated DSB end-resection and subsequent HR-mediated DSB repair. None the less, as pointed out by Lu and Hunter,^38^ some skepticism of PKM2’s function as a protein kinase must remain until such time that a crystal structure of PKM2 bound to a substrate is achieved to clearly demonstrate how a target residue in the substrate is presented to the active site in PKM2 to accommodate phosphate transfer.

CtIP activity is regulated by a variety of kinases involved in the DNA damage response, suggesting maximal CtIP activity requires positive input from multiple pathways.^39^ One example of the coordinated activation of CtIP by multiple inputs is the finding by Wang et al. that CDK-dependent phosphorylation of CtIP is prerequisite for phosphorylation of CtIP by ATM and maximal end-resection following DNA damage.^19^ Our results suggest that maximal activation of CtIP in the models utilized by this study requires PKM2 activation by ATM. ATM-regulated PKM2-mediated activation of CtIP may represent a means for highly proliferative cancer cells to accentuate this repair mechanism and may be one of the important mechanisms through which these cells manage increased oxidative stress. How phosphorylation of T126 influences other posttranslational modifications of CtIP will be the focus of future studies.

PKM2 is a cytoplasmic–nuclear shuttling protein, and the regulatory mechanisms for PKM2 trafficking have been well characterized^40^ beginning with Yang et al.’s demonstration that phosphorylation of S37 on PKM2 by ERK1/2 is required for translocation of PKM2 to the nucleus in response to EGFR signaling.^6^ A number of additional post-translational modifications to PKM2, including SUMOylation by PIAS3^41^ and acetylation by the p300 acetyltransferase^42^, have also been demonstrated to facilitate its nuclear translocation. We show that phosphorylation of PKM2 T328 by ATM occurs in the nucleus and is critical for PKM2’s nuclear retention following DNA damage. Nuclear retention of PKM2 is required for its interaction with and activation of CtIP, although the absolute requirement of pT328-PKM2 for this interaction cannot yet be concluded. Whether other mechanisms are activated following DNA damage that increase PKM2 transport into the nucleus, the precise mechanisms through which phosphorylation of PKM2 on T328 keeps PKM2 in the nucleus, and the detailed mechanism of PKM2-CtIP interaction will be the subject of future studies.

Recently, PKM2 was found to be phosphorylated on T328 by GSK-3β and this phosphorylation was critical for the stability and biological function of PKM2 in hepatocellular carcinoma.^43^ GSK3β is a cytoplasmic-nuclear shuttling protein that is active in the nucleus following DNA damage.^44^ Therefore, we cannot rule out that in addition to ATM, GSK3β may also phosphorylate nuclear PKM2 in response to DNA damage. However, active GSK3β generally promotes cell death rather than DNA repair, whereas inhibition of GSk3β enhances DSB repair.^45^ Furthermore, activity of nuclear GSK3β was recently shown to be attenuated by ATM through p38 to promote repair of DSBs.^46^ As such, it is unlikely that GSK3β plays a significant role, relative to ATM, in phosphorylation of T328 PKM2 following DNA damage.

**Fig. 7:**
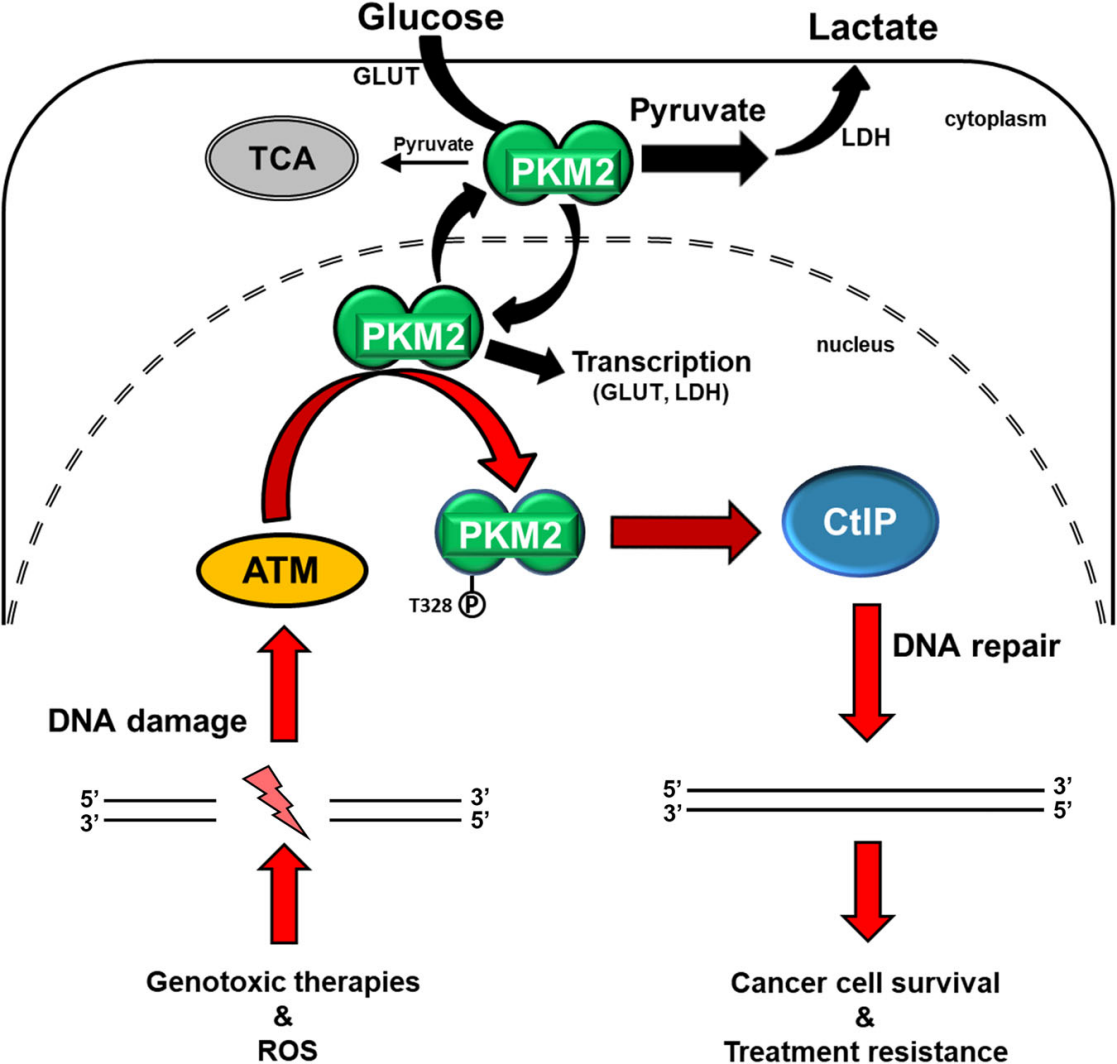
PKM2 is over-expressed in cancer where it unites cancer glycolytic metabolism with DNA repair to drive treatment resistance. PKM2 is frequently overexpressed in cancer cells where cytosolic PKM2 reprograms glucose metabolism (Warburg effect), while nuclear PKM2 has additional oncogenic function as a transcriptional coactivator (black arrows). Our data uncovered a novel nuclear function of PKM2 through which it directly regulates DNA DSB repair and resistance to genotoxic damage (red arrows). ATM, activated by DNA DSBs, phosphorylates nuclear PKM2 at T328, resulting in nuclear retention and accumulation of PKM2. pT328-PKM2 interacts with CtIP and phosphorylates CtIP on T126 to increase its function in HR repair, resulting in increased DSB repair efficiency and resistance to genotoxic treatments

Overexpression of PKM2 is a trait shared by the majority of cancers and this PK isoform is critical for cancer metabolic reprograming via its functions as a pyruvate kinase in the cytosol and a protein kinase and transcriptional coactivator in the nucleus.^40^ Our data revealed an unexpected role of nuclear PKM2 in promoting HR-mediated repair of DNA damage, positioning PKM2 at the convergence of metabolic reprograming and DNA repair (Fig. 7). The findings presented herein promote the development of PKM2-targeted therapies not only as a means to target cancer metabolism, but more importantly to inhibit an essential mechanism of cancer cell resistance to genotoxic damage. Strategies that combine PKM2 inhibition with genotoxic agents may more effectively take advantage of this apparent Achilles’ heel in cancer.

## Materials and methods

### Cell survival assays

Cell survival was analyzed by colony formation as described previously^47^ or by trypan blue staining of viable cells.

### In vivo tumor models

All animal procedures were approved by the Institutional Animal Care and Use Committee at The Ohio State University. U87 cells (2.5 × 10^6^) expressing shCtrl and shPKM2 were subcutaneously injected into the flanks of 6–8 week old female athymic nu/nu mice (Charles River). Mice were randomly assigned to control or radiation treatment (2 Gy × 5 consecutive days) arms when the volume of their tumor exceeded 100 mm^3^. Tumor volume was monitored until removal criteria for the study were reached, at which point the animal was sacrificed. Survival was studied in mice bearing intracranial U87/EGFRvIII tumors expressing shCtrl or shPKM2, which grew from 50,000 cells stereotactically implanted into the brains of 6–8 week old female athymic nu/nu mice. Mice were imaged by MRI on days 8 and 15 after implantation to monitor the presence of tumors. On day 15, mice with confirmed intracranial tumors were randomly assigned to control or radiation treatment (4 Gy × 3 consecutive days) groups. Mice were sacrificed when they became moribund.

### Nonhomologous end-joining (NHEJ) repair assay

The system used to measure NHEJ repair of I-SceI-induced DSBs has been described previously.^17^ Briefly, pEJ is an NHEJ-dependent GFP-reporter assay stably integrated into the genome of the U87 cells. GFP translation is prevented in this reporter by the presence of an out-of-frame ATG in the 5′-untranslated region between the CMV promoter and the GFP open reading frame. Two repeated I-SceI sites in inverted orientation flank this out-of-frame ATG. Cleavage by I-SceI removes the out-of-frame ATG and creates non-cohesive double-stranded ends. Repair of this DSB by NHEJ results in GFP translation, which was measured by flow cytometry (FACSort, Becton-Dickinson).

### Homologous recombination repair assay

The DR-GFP reporter system used to determine repair of I-SceI-induced DSBs via HR has been described previously.^18^ We transfected the DR-GFP plasmid into human U87 glioma cells and established a stably transfected clone by selection with puromycin (5 μg/mL). Briefly, this reporter contains a cassette of two differently mutated GFP genes in direct repeat. One repeat contains a single I-SceI site, while the other copy is a truncated GFP fragment. I-SceI generates a DSB that, if repaired by HR, results in a complete GFP open reading frame. Translation of GFP was measured by flow cytometry (FACSort, Becton-Dickinson).

### CtIP chromatin immunoprecipitation

HT1904 cells containing the I-SceI DSB system and its use to analyze protein binding upstream of I-SceI-induced DSBs have been described previously.^48^ ChIP was performed per the manufacturer’s recommendations using a Chromatin Immunoprecipitation Assay Kit (Millipore), anti-CtIP antibody (Active Motif) and primers targeting sites 604, 152, and 50 bp upstream from the I-SceI site (see Supplemental information, Data S1).

### Statistical analyses

Statistical analyses were performed using Winstat or Excel. Unless otherwise noted, statistical analysis between groups was performed by ANOVA and direct comparisons between two groups were done by a two-tailed *t*-test. *P* < 0.05 was considered statistically significant.

## Electronic supplementary material

Supplementary information, Figure S1

Supplementary information, Figure S2

Supplementary information, Figure S3

Supplementary information, Figure S4

Supplementary information, Figure S5

Supplementary information, Figure S6

Supplementary information, Figure S7

Supplementary information, Figure S8

Supplementary information, Figure S9

Supplementary information, Data S1
