# Supplementary material for: Pyruvate kinase M2 regulates homologous recombination-mediated DNA double-strand break repair
Source: Cell Res. 2018 Oct 8;28(11):1090–102. doi: 10.1038/s41422-018-0086-7 (PMC6218445; doi:10.1038/s41422-018-0086-7)
Supplement: Supplementary file 1 — Supplementary information, Figure S1 [file 41422_2018_86_MOESM1_ESM.pdf]

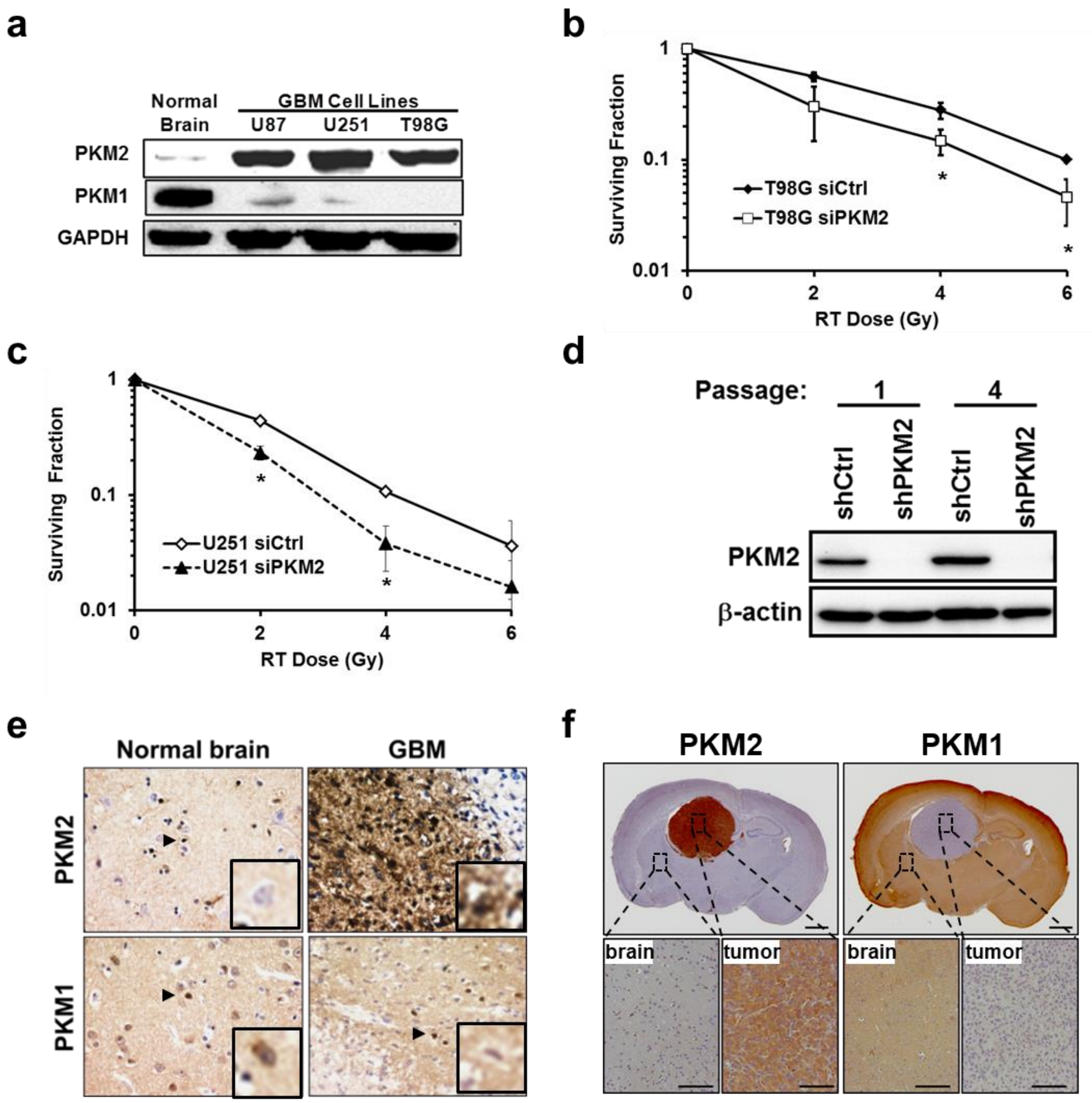

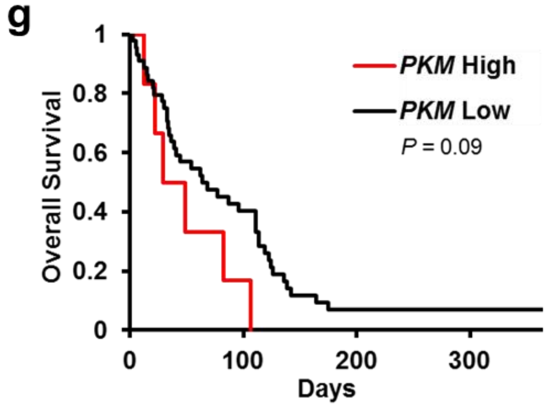

Number of patients remaining at risk					
	Day				
Group	0	100	200	300	365
PKM2 High	6	1	0	0	0
PKM2 Low	45	18	4	3	3

Figure S1. a Western blot analysis of PKM1 and PKM2 in normal brain tissue and U87, U251, and T98G GBM cell lines. GAPDH served as the loading control. **b** Clonogenic survival of T98G cells treated with control or PKM2 siRNA 24 h prior to irradiation as indicated. RT, radiation therapy. Results are mean ± SD (n = 3). **c** Clonogenic survival of U251 cells treated with control or PKM2 siRNA 24 h prior to irradiation. Cells were irradiated with 0, 2, 4, or 6 Gy as indicated. RT, radiation therapy. Results are mean ± SD (n = 2). **d** Western blot analysis of PKM2 expression in stable U87/EGFRvIII shCtrl and shPKM2 cells at 1 and 4 passages after puromycin selection. β-actin served as the loading control. **e** Immunohistochemical staining for PKM2 (top) and PKM1 (bottom) in representative normal human brain and GBM sections. Inserts show staining in representative cells. Arrowheads indicate lymphocytes that stain for both PKM1 and PKM2. **f** Representative low magnification (upper panels, scale bar = 1 mm) and high magnification (bottom panels, scale bar = 0.1 mm) images showing immunohistochemical staining for PKM2 (left) and PKM1 (right) in representative sections of a mouse brain containing a U87/EGFRvIII intracranial tumor. **g** Patients in the TCGA GBM cohort receiving no genotoxic treatment were stratified by *PKM* expression (*PKM* High = upper 10th percentile; *PKM* Low = lower 90th percentile) and overall survival was analyzed by the Kaplan Meier method ($P = 0.09$ for the difference between high and low expression groups). *, $P < 0.05$.
