# Supplementary material for: Pyruvate kinase M2 regulates homologous recombination-mediated DNA double-strand break repair
Source: Cell Res. 2018 Oct 8;28(11):1090–102. doi: 10.1038/s41422-018-0086-7 (PMC6218445; doi:10.1038/s41422-018-0086-7)
Supplement: Supplementary file 2 — Supplementary information, Figure S2 [file 41422_2018_86_MOESM2_ESM.pdf]

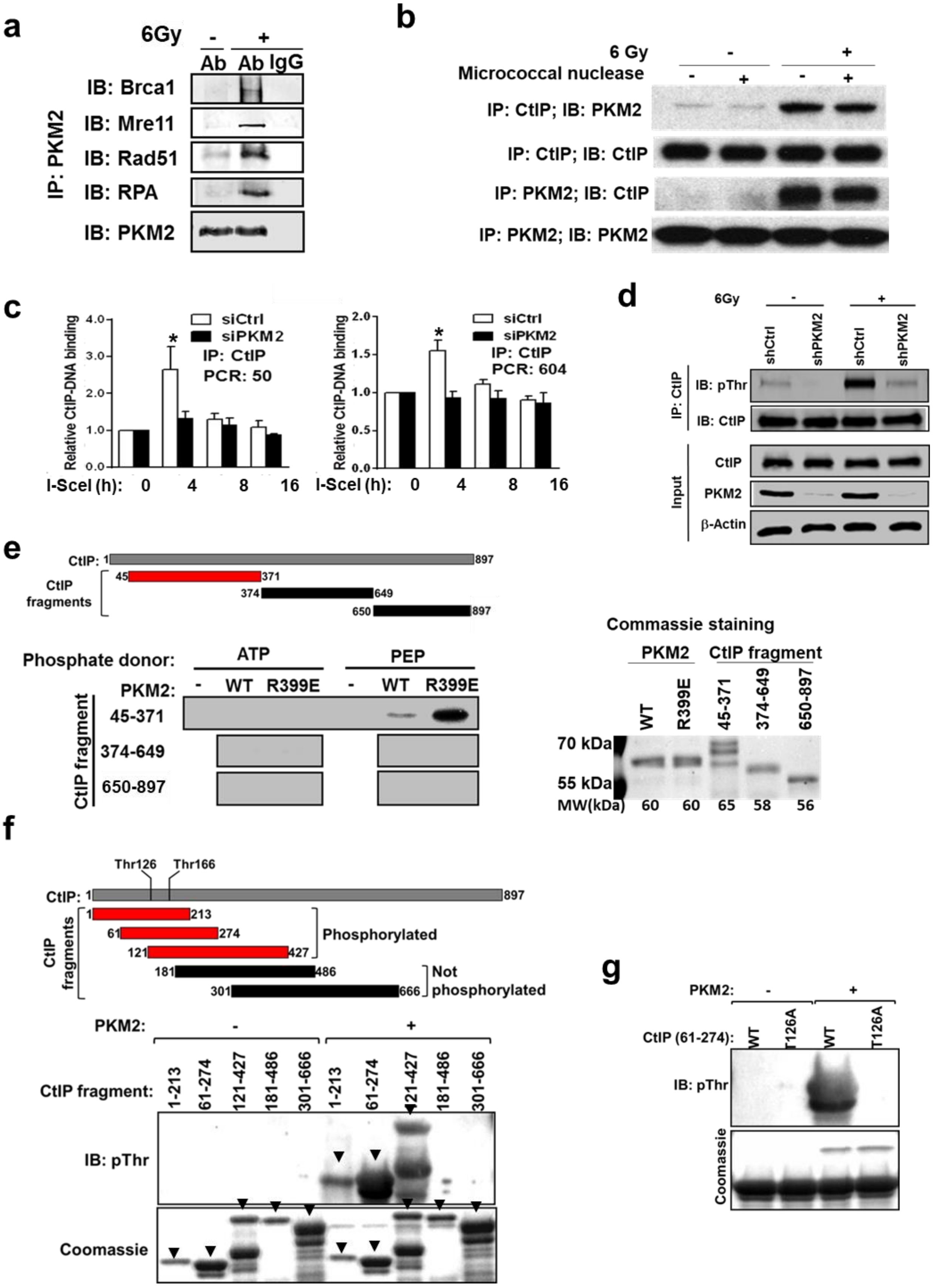
a
6Gy
-
+
Ab
Ab
IgG
IP: PKM2
IB: Brca1
IB: Mre11
IB: Rad51
IB: RPA
IB: PKM2
b
6 Gy
Micrococcal nuclease
IP: CtIP; IB: PKM2
IP: CtIP; IB: CtIP
IP: PKM2; IB: CtIP
IP: PKM2; IB: PKM2
c
Relative CtIP-DNA binding
siCtrl
siPKM2
IP: CtIP
PCR: 50
PCR: 604
I-SceI (h):
0
4
8
16
d
6Gy
shCtrl
shPKM2
IP: CtIP
IB: pThr
IB: CtIP
Input
CtIP
PKM2
β-Actin
e
CtIP:
CtIP fragments
Phosphate donor:
ATP
PEP
PKM2:
WT
R399E
CtIP fragment
45-371
374-649
650-897
Commassie staining
PKM2
CtIP fragment
70 kDa
55 kDa
MW(kDa)
60
60
65
58
56
f
Thr126
Thr166
CtIP:
CtIP fragments
Phosphorylated
Not phosphorylated
PKM2:
CtIP fragment:
1-213
61-274
121-427
181-486
301-666
IB: pThr
Coomassie
g
PKM2:
CtIP (61-274):
WT
T126A
IB: pThr
Coomassie

Figure S2. **a** Protein lysates were collected from U87 cells after irradiation and immunoprecipitated with PKM2 antibody or anti-rabbit IgG. Immunoprecipitates were separated by SDS-PAGE and immunoblotted with the indicated antibodies. **b** Protein lysates from unirradiated or irradiated U87 cells were collected, treated with micrococcal nuclease and immunoprecipitated with PKM2 and CtIP antibodies. Immunoprecipitates were analyzed by western blot. **c** HT1904 cells containing the I-SceI DSB system were utilized to assess CtIP binding 50 bp (left panel) and 604 bp (right panel) upstream of I-SceI-induced DSBs. Cells were transfected with control or PKM2-targeting siRNA. After 24 h, I-SceI adenovirus was introduced to create DSBs. At the indicated time points, CtIP-DNA complexes were harvested and CtIP binding was quantified by real-time PCR. Data are normalized to T=0 and presented as mean ± SE (n = 3). **d** CtIP was immunoprecipitated from U87 shCtrl and shPKM2 cells treated with 0 or 6 Gy radiation. Immunoprecipitates were separated by SDS-PAGE and immunoblotted with antibodies to phosphothreonine and CtIP. Input protein lysates were also separated by SDS-PAGE and immunoblotted for CtIP, PKM2, or β-actin as a loading control. **e** A schematic illustrating the recombinant CtIP peptide fragments utilized as substrate for *in vitro* PKM2 kinase reactions. Kinase reactions were performed using either ATP or PEP as phosphate donor and in the absence (-) or presence of wild-type PKM2 (WT) or R399E-PKM2. Reactions were separated by SDS-PAGE and immunoblotted with anti-phosphothreonine antibody. **f** A schematic illustrating the recombinant CtIP peptide fragments utilized as substrate for *in vitro* PKM2 kinase reactions. Kinase reactions in the absence (-) or presence (+) of PKM2 were separated by SDS-PAGE and immunoblotted with anti-phosphothreonine antibody or stained with Coomassie**.** Arrowheads mark full-

length recombinant CtIP fragments. **g** CtIP fragment 61-274 (WT) or this fragment carrying a T126A mutation were utilized in kinase reactions in the absence (-) or presence of PKM2 (+). Kinase reactions were separated by SDS-PAGE and immunoblotted with anti-phosphothreonine antibody or stained with Coomassie.
