# Supplementary material for: Pyruvate kinase M2 regulates homologous recombination-mediated DNA double-strand break repair
Source: Cell Res. 2018 Oct 8;28(11):1090–102. doi: 10.1038/s41422-018-0086-7 (PMC6218445; doi:10.1038/s41422-018-0086-7)
Supplement: Supplementary file 3 — Supplementary information, Figure S3 [file 41422_2018_86_MOESM3_ESM.pdf]

a

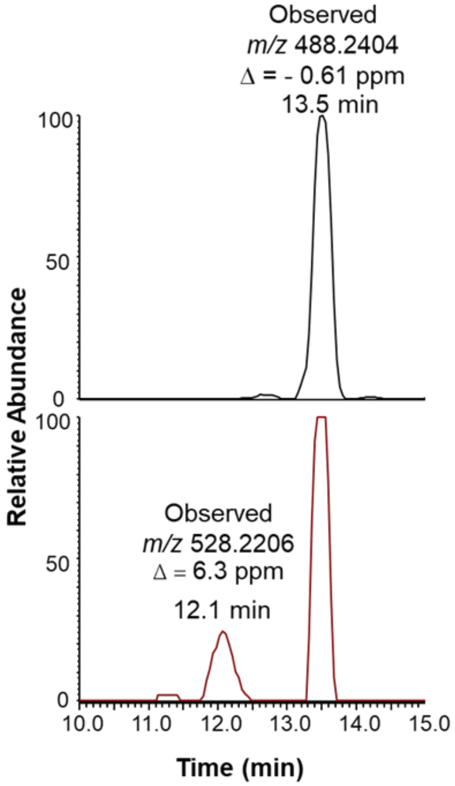
Observed
m/z 488.2404
Δ = - 0.61 ppm
13.5 min
Observed
m/z 528.2206
Δ = 6.3 ppm
12.1 min
Relative Abundance
100
50
0
10.0 11.0 12.0 13.0 14.0 15.0
Time (min)

b

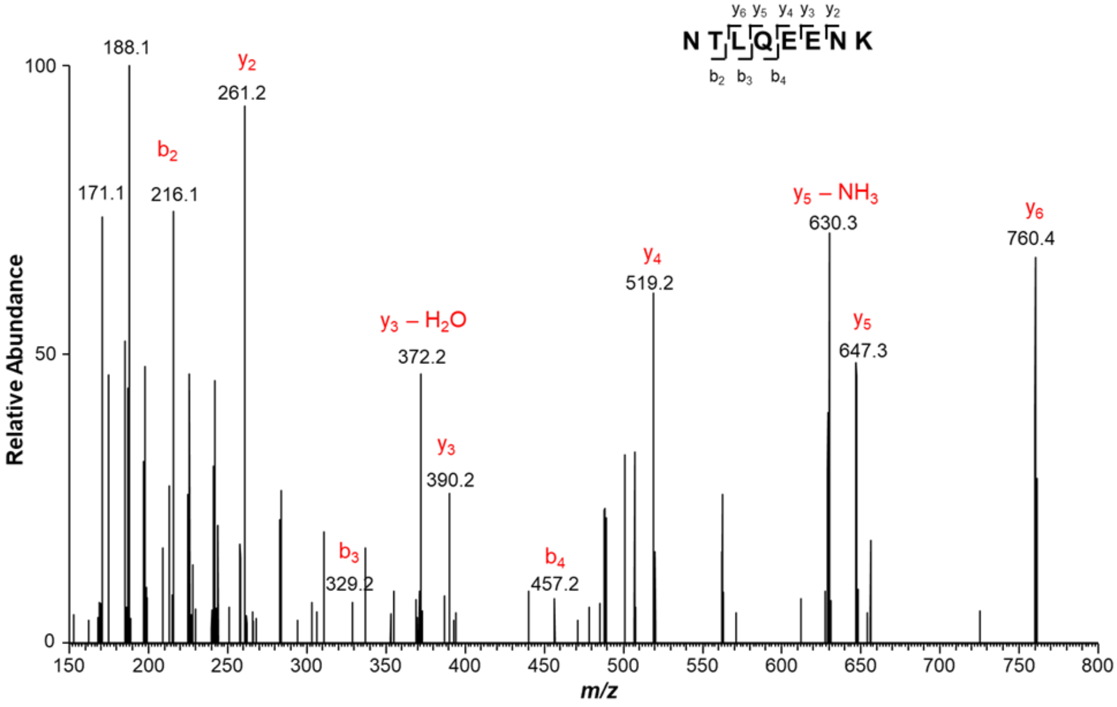
N T L Q E E N K
y6 y5 y4 y3 y2
b2 b3 b4
188.1
y2 261.2
b2 216.1
171.1
y5 – NH3 630.3
y6 760.4
y4 519.2
y3 – H2O 372.2
y5 647.3
y3 390.2
b3 329.2
b4 457.2
Relative Abundance
100
50
0
150 200 250 300 350 400 450 500 550 600 650 700 750 800
m/z

Figure S3. LC-MS and MS/MS for the identification of the unmodified CtIP peptide, NTLQEENK and the same peptide with Thr^{126} being phosphorylated from the irradiated sample. **a** Selected-ion chromatogram for the $[M + 2H]^{2+}$ ions of the unphosphorylated (top, calculated *m/z* 488.2407) and phosphorylated (bottom, calculated *m/z* 528.2239) peptide NTLQEENK. **b** Tandem mass spectrum (MS/MS) of the ESI-produced $[M+2H]^{2+}$ ion of the unphosphorylated peptide NTLQEENK. The spectrum was acquired by LC-MS/MS in the data-dependent acquisition mode on a QE Plus quadrupole-Orbitrap mass spectrometer.
