# Supplementary material for: Pyruvate kinase M2 regulates homologous recombination-mediated DNA double-strand break repair
Source: Cell Res. 2018 Oct 8;28(11):1090–102. doi: 10.1038/s41422-018-0086-7 (PMC6218445; doi:10.1038/s41422-018-0086-7)
Supplement: Supplementary file 4 — Supplementary information, Figure S4 [file 41422_2018_86_MOESM4_ESM.pdf]

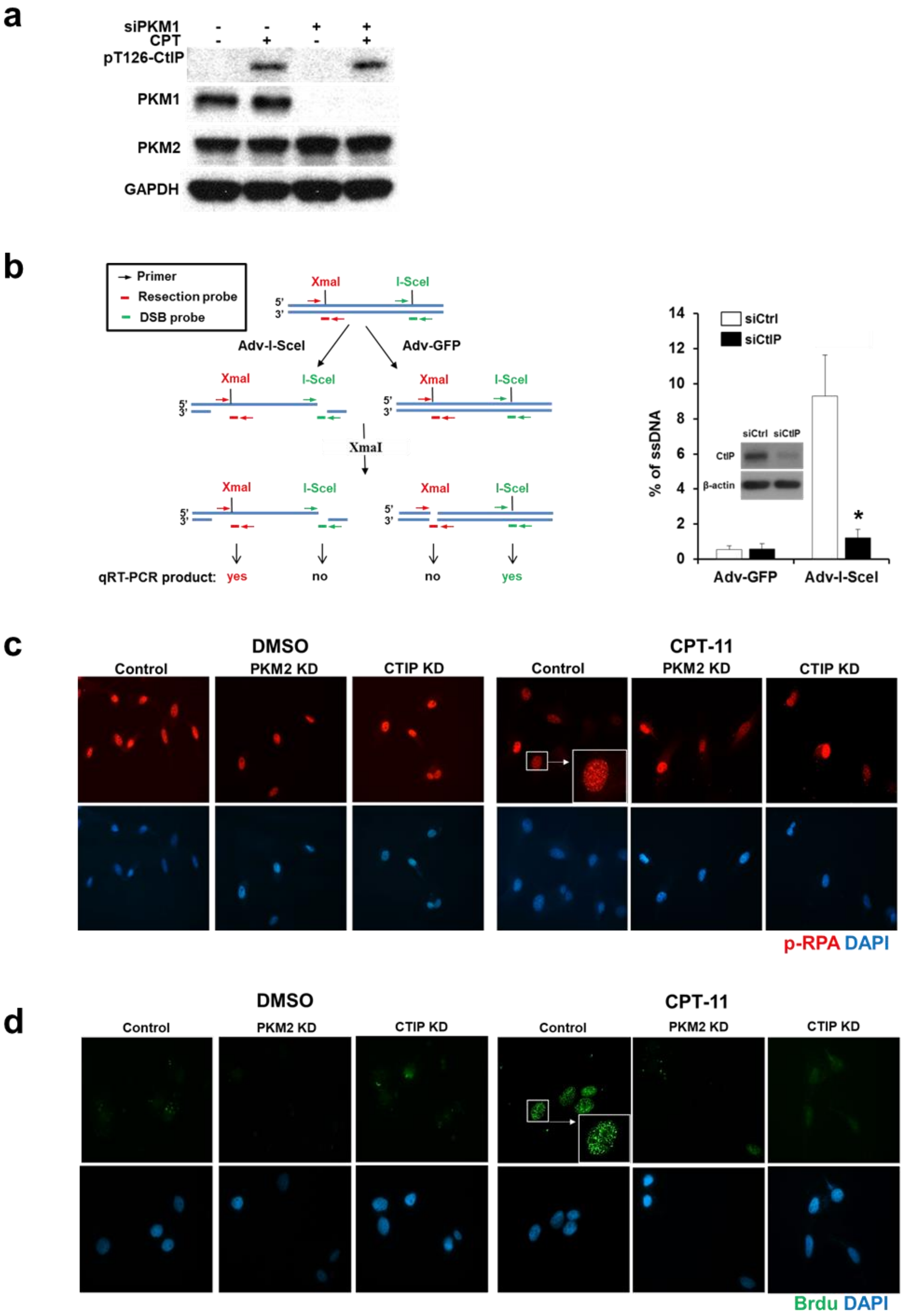
a
siPKM1 - - + +
CPT - + - +
pT126-CtIP
PKM1
PKM2
GAPDH
b
Primer
Resection probe
DSB probe
XmaI
I-SceI
5'
3'
Adv-I-SceI
Adv-GFP
XmaI
qRT-PCR product: yes no no yes
siCtrl
siCtIP
% of ssDNA
14
12
10
8
6
4
2
0
CtIP
β-actin
*
Adv-GFP
Adv-I-SceI
c
DMSO
CPT-11
Control
PKM2 KD
CTIP KD
p-RPA DAPI
d
DMSO
CPT-11
Control
PKM2 KD
CTIP KD
Brdu DAPI

Figure S4. **a** U87 cells were first treated with 200nM siRNA to deplete PKM1 and 48h later treated with 1μM CPT-11 to induce DNA damage. Cell lysates were collected 1h later and analyzed for pT126-CtIP. **b** Schematic of the resection assay utilizing HT1904 cells containing the I-SceI DSB system. A quantitative real-time PCR primer/probe set was used to measure DSBs generated by I-SceI (green), while another primer/probe set (red) was used to measure the presence of ssDNA extending beyond an XmaI site 328bp from the I-SceI site. XmaI cannot cut ssDNA. Knockdown of CtIP in this system significantly reduced resection of DNA adjacent to I-SceI-induced DSBs. Data are presented as mean ± SE (n = 3). *, $P < 0.05$. **c** Representative images of DMSO or CTP-11 treated U87 control, PKM2 knockdown, or CtIP knockdown cells stained for RPA (red). Nuclei are stained with DAPI (Blue). **d** Representative images of DMSO or CTP-11 treated U87 control, PKM2 knockdown, or CtIP knockdown cells stained for BrdU (red). Nuclei are stained with DAPI (Blue).
