# Supplementary material for: Pyruvate kinase M2 regulates homologous recombination-mediated DNA double-strand break repair
Source: Cell Res. 2018 Oct 8;28(11):1090–102. doi: 10.1038/s41422-018-0086-7 (PMC6218445; doi:10.1038/s41422-018-0086-7)
Supplement: Supplementary file 5 — Supplementary information, Figure S5 [file 41422_2018_86_MOESM5_ESM.pdf]

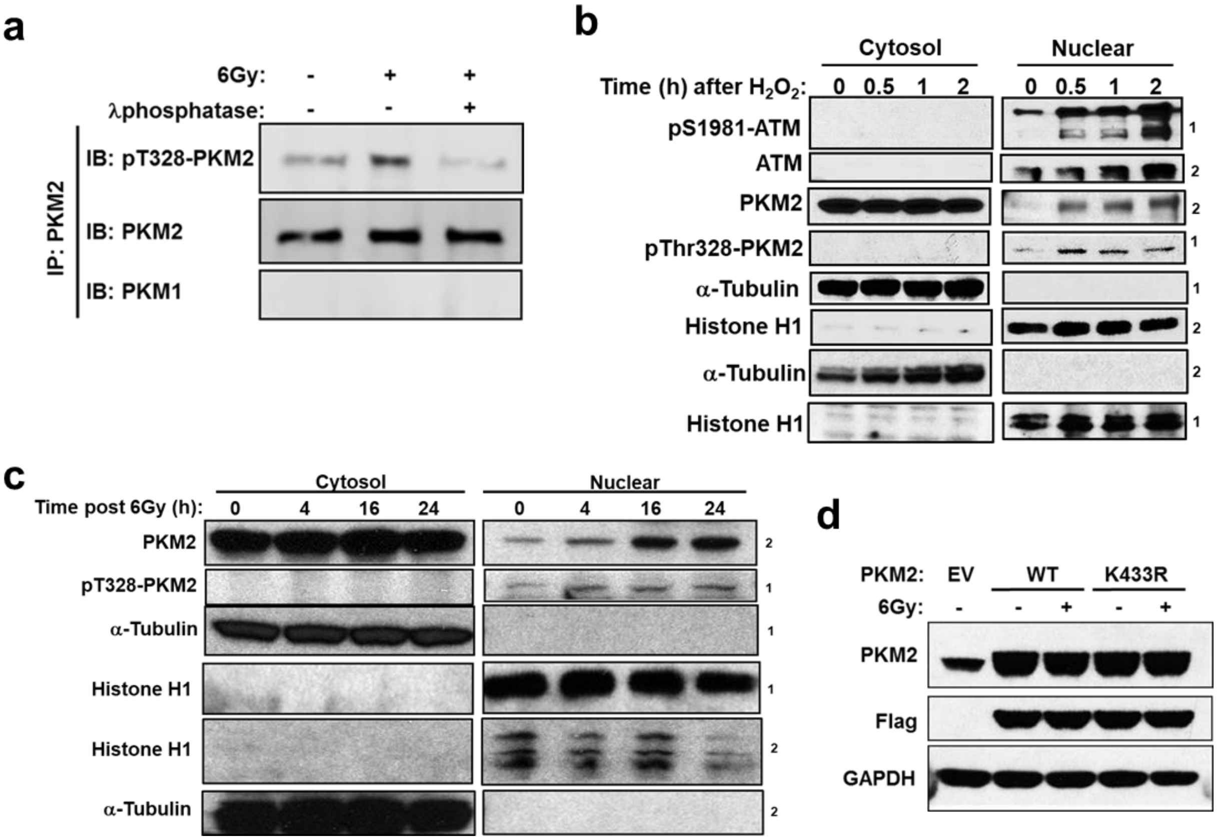

Figure S5. **a** Protein lysates from U87 cells treated with 0 or 6 Gy were incubated with λ- phosphatase as indicated and then immunoprecipitated with PKM2 antibody. The immunoprecipitates were separated by SDS-PAGE and immunoblotted with antibodies to pT328-PKM2, PKM2, and PKM1. **b** U87 cells were treated with 0.5 mM H_2O_2 and cell lysates were collected at the indicated times and separated into nuclear and cytoplasmic fractions which were resolved by SDS-PAGE and probed with the indicated antibodies. Superscripts [1] and [2] identify respective membranes used for immunoblotting. **c** U87 cells were treated with 6Gy radiation and cell lysates were collected at the indicated times and separated into nuclear and cytoplasmic fractions which were resolved by SDS-PAGE and probed with the indicated antibodies. Superscripts [1] and [2] identify respective membranes used for immunoblotting. **d** Representative western blot analyses of PKM2 and flag

expression in U87 cells transfected with empty vector (EV) or vector encoding WT or K433R PKM2. Cells were treated with 6 Gy radiation as indicated. GAPDH served as the loading control.
