# Supplementary material for: Pyruvate kinase M2 regulates homologous recombination-mediated DNA double-strand break repair
Source: Cell Res. 2018 Oct 8;28(11):1090–102. doi: 10.1038/s41422-018-0086-7 (PMC6218445; doi:10.1038/s41422-018-0086-7)
Supplement: Supplementary file 6 — Supplementary information, Figure S6 [file 41422_2018_86_MOESM6_ESM.pdf]

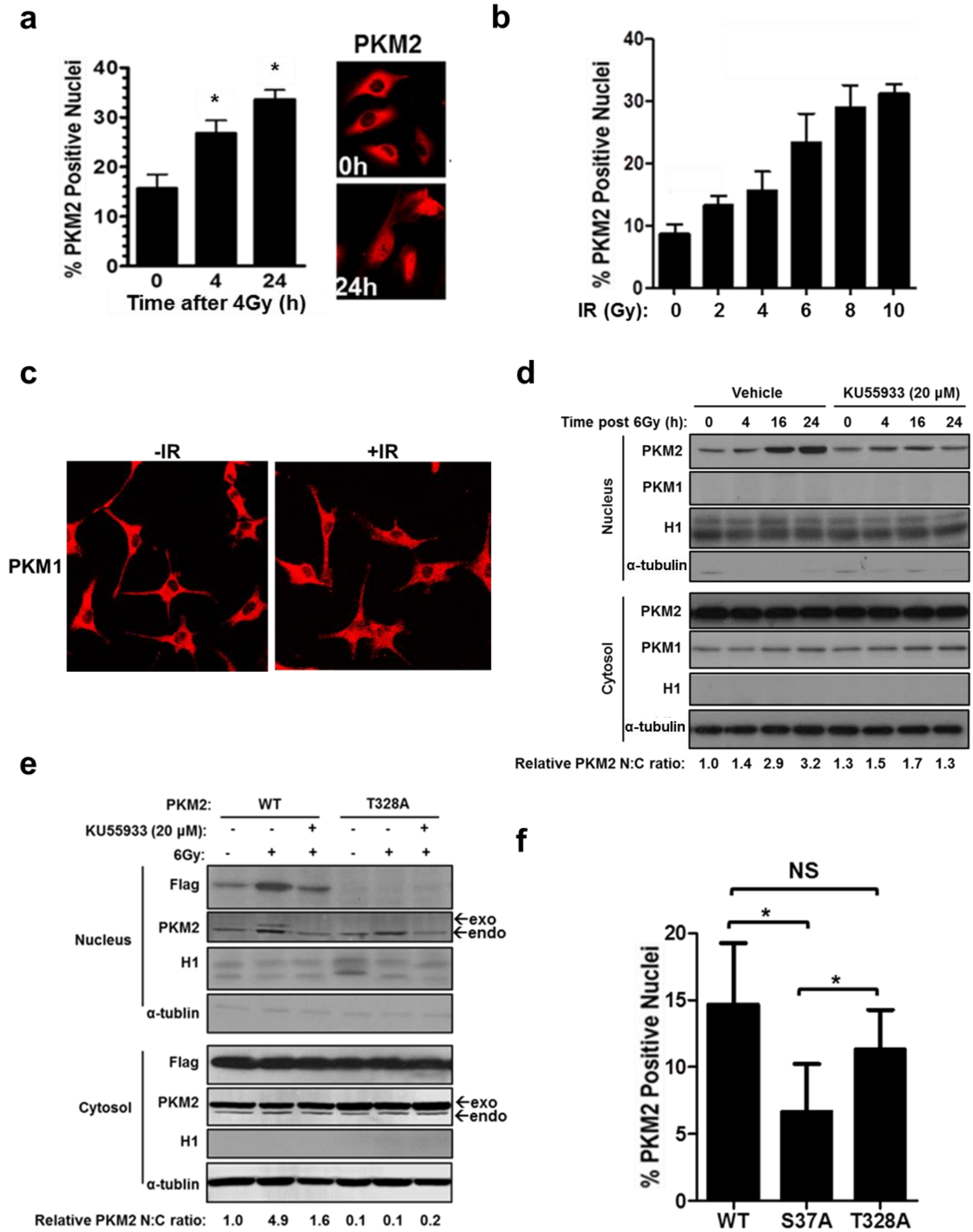
a
PKM2
% PKM2 Positive Nuclei
40
30
20
10
0
*
*
0
4
24
Time after 4Gy (h)
0h
24h
b
% PKM2 Positive Nuclei
40
30
20
10
0
IR (Gy): 0 2 4 6 8 10
c
-IR
+IR
PKM1
d
Vehicle
KU55933 (20 μM)
Time post 6Gy (h): 0 4 16 24 0 4 16 24
Nucleus
PKM2
PKM1
H1
α-tubulin
Cytosol
PKM2
PKM1
H1
α-tubulin
Relative PKM2 N:C ratio: 1.0 1.4 2.9 3.2 1.3 1.5 1.7 1.3
e
PKM2: WT T328A
KU55933 (20 μM): - - + - - +
6Gy: - + + - + +
Nucleus
Flag
PKM2
←exo
←endo
H1
α-tublin
Cytosol
Flag
PKM2
←exo
←endo
H1
α-tublin
Relative PKM2 N:C ratio: 1.0 4.9 1.6 0.1 0.1 0.2
f
NS
*
*
% PKM2 Positive Nuclei
20
15
10
5
0
WT
S37A
T328A

Figure S6. a U87 cells were irradiated and then fixed at the indicated time points, and PKM2 localization was determined by immunohistochemical staining. Data are the mean percentage of cells with nuclear PKM2 staining ± SE (n = 3). Photos represent typical PKM2 cytoplasmic staining seen at 0 h and increased nuclear PKM2 staining observed 24 h post-radiation. **b** U87 cells were irradiated with the indicated doses, fixed, and PKM2 subcellular localization was determined by immunohistochemical staining. Data are the mean percentage of cells with nuclear PKM2 staining ± SE (n = 3). **c** Representative images of immunohistochemical staining for PKM1 in U87 cells in the absence of radiation (left) or 24 h after treatment with 6 Gy (right). **d** U87 cells were treated with DMSO or KU55933 (20 μM) and then subjected to radiation. Lysates were harvested at the indicated times and separated into nuclear and cytoplasmic fractions, which were resolved by SDS-PAGE and probed with antibodies to PKM2 and PKM1. Histone H1 served as the nuclear protein loading control while α-tubulin was the cytoplasmic protein control. The relative ratio of nuclear (N) to cytoplasmic (C) PKM2 was quantitated using ImageJ software. **e** U87 shPKM2 cells expressing shRNA resistant, flag-tagged WT or T328A PKM2 were pre-treated with DMSO or KU55933 (20 μM) and then irradiated. Cell lysates were collected and separated into nuclear and cytoplasmic fractions, which were resolved by SDS-PAGE and probed with antibodies to flag and PKM2. The relative ratio of nuclear (N) to cytoplasmic (C) PKM2 was quantitated using ImageJ software. **f** U87 shPKM2 cells expressing WT, S37A, or T328A PKM2 were treated with EGF (100 ng/mL), fixed, and PKM2 nuclear localization was determined by immunohistochemical staining. Data are presented as the mean percentage of cells with nuclear PKM2 staining ± SE (n = 3). *, P <0.05. NS, not significant.
