# Supplementary material for: Pyruvate kinase M2 regulates homologous recombination-mediated DNA double-strand break repair
Source: Cell Res. 2018 Oct 8;28(11):1090–102. doi: 10.1038/s41422-018-0086-7 (PMC6218445; doi:10.1038/s41422-018-0086-7)
Supplement: Supplementary file 7 — Supplementary information, Figure S7 [file 41422_2018_86_MOESM7_ESM.pdf]

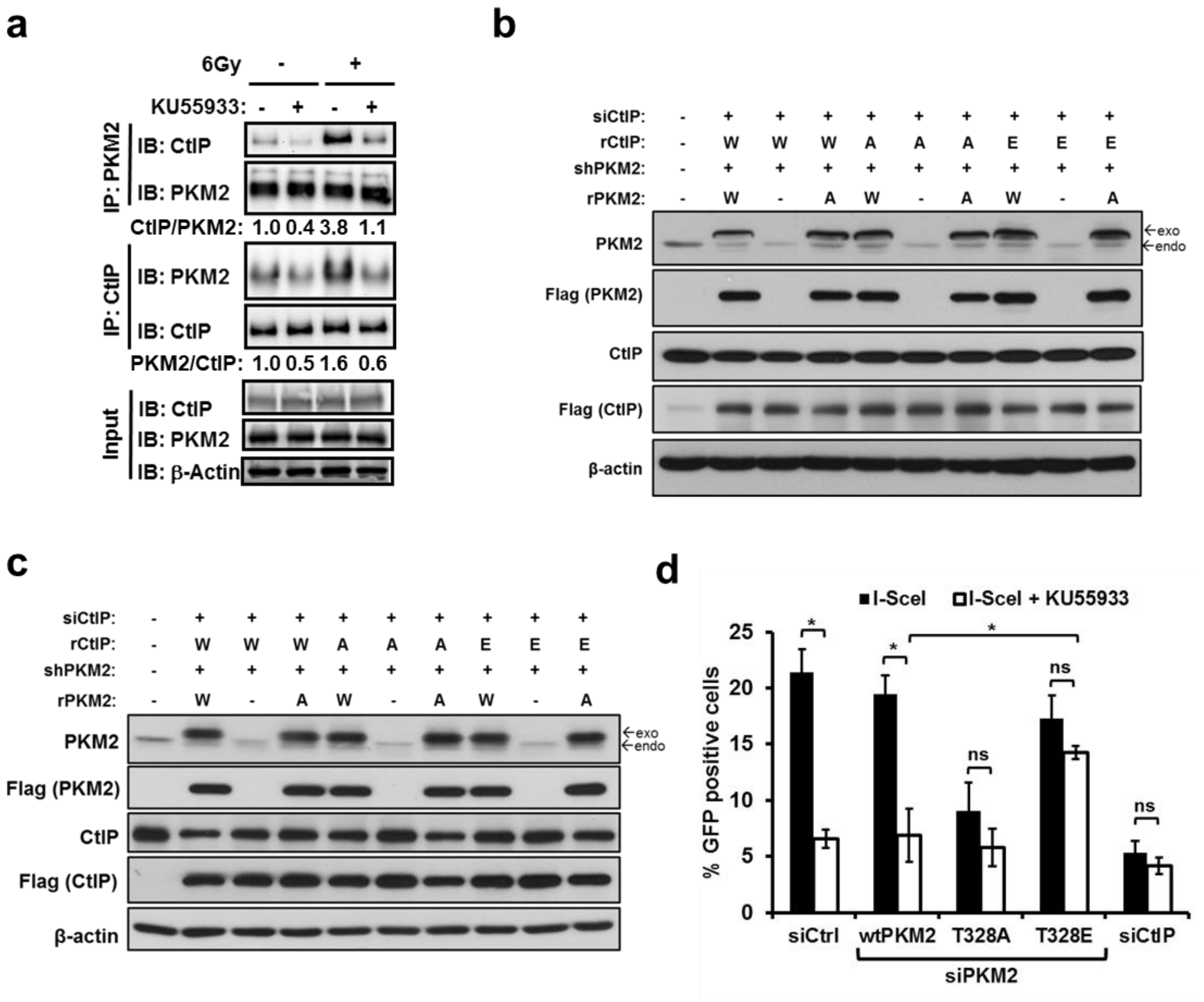

Figure S7. a U87 cells were treated with DMSO or KU55933 (20 µM), then subjected to radiation. Cell lysates were collected and immunoprecipitated with PKM2 or CtIP antibodies. Immunoprecipitates and input lysates were separated by SDS-PAGE and probed with the specified antibodies. The relative ratio of CtIP to PKM2 band intensities was analyzed using ImageJ software. **b** and **c** Representative western blot analyses of PKM2, flag-tagged PKM2, CtIP, and flag-tagged CtIP from **b** HT1904 cells utilized for DNA end-resection analyses or **c** U87 DR-GFP cells utilized in HR repair analyses. PKM2 and CtIP were knocked down and replaced by WT or T328A PKM2 (A) and WT, T126A

(A), or T126E CtIP (E) as shown. β-actin served as the loading control. **d** PKM2 was knocked down in U87 DR-GFP cells and replaced by WT, T328A, or T328E PKM2 as indicated. CtIP was knocked down in these cells as an additional control. After 24 h, the cells were treated with DMSO or 20 μM KU55933 prior to adenoviral delivery of I-SceI. Resolution of I-SceI-induced DSBs by the HR pathway was assessed by measuring the percentage of GFP-positive cells 48 h later. Data are presented as mean ± SE (n = 3). *, $P < 0.05$. ns, not significant.
