# Supplementary material for: Pyruvate kinase M2 regulates homologous recombination-mediated DNA double-strand break repair
Source: Cell Res. 2018 Oct 8;28(11):1090–102. doi: 10.1038/s41422-018-0086-7 (PMC6218445; doi:10.1038/s41422-018-0086-7)
Supplement: Supplementary file 8 — Supplementary information, Figure S8 [file 41422_2018_86_MOESM8_ESM.pdf]

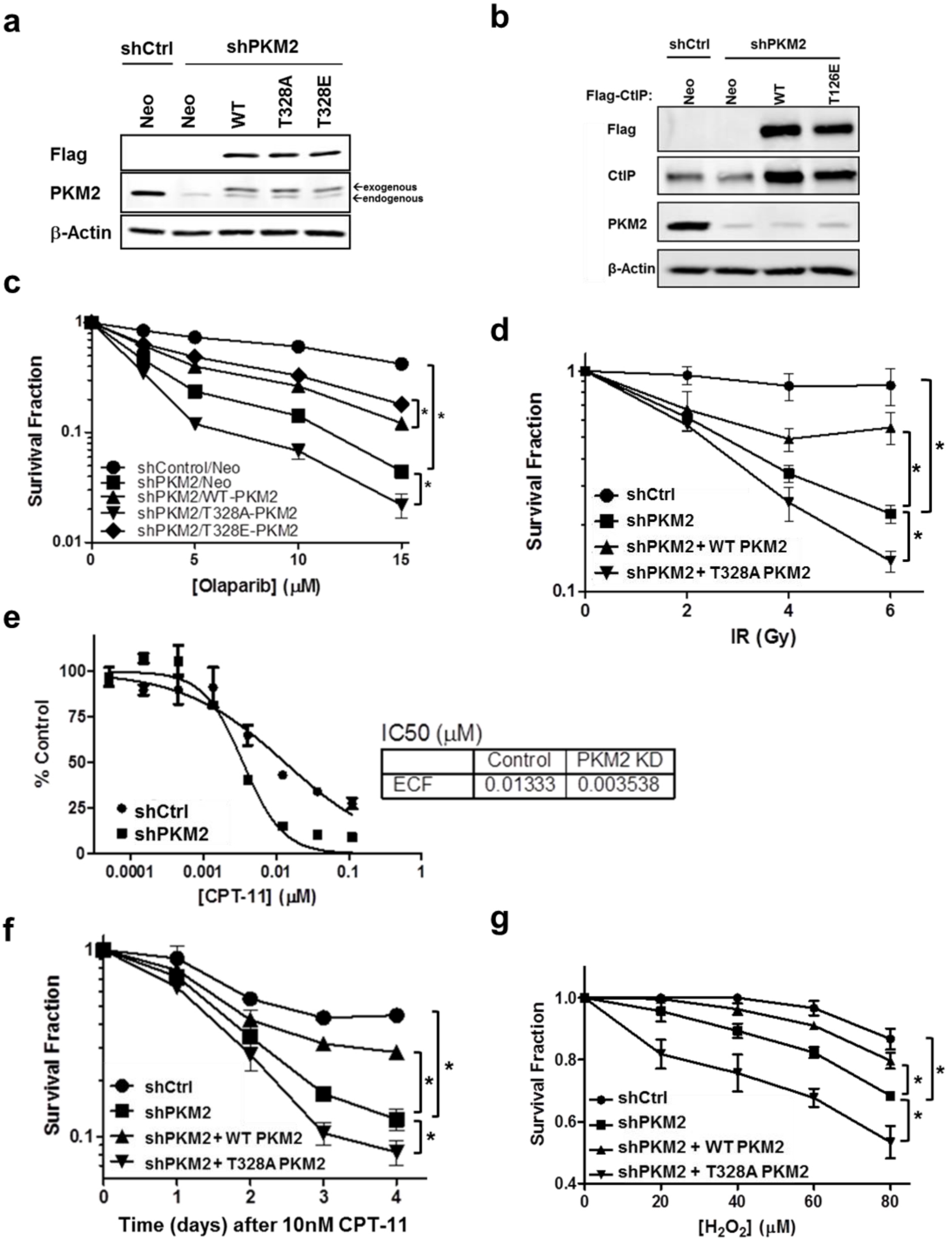
a
shCtrl
shPKM2
Neo
Neo
WT
T328A
T328E
Flag
PKM2
β-Actin
←exogenous
←endogenous
b
shCtrl
shPKM2
Flag-CtIP:
Neo
Neo
WT
T126E
Flag
CtIP
PKM2
β-Actin
c
Surivival Fraction
[Olaparib] (μM)
shControl/Neo
shPKM2/Neo
shPKM2/WT-PKM2
shPKM2/T328A-PKM2
shPKM2/T328E-PKM2
d
Survival Fraction
IR (Gy)
shCtrl
shPKM2
shPKM2+ WT PKM2
shPKM2+ T328A PKM2
e
% Control
[CPT-11] (μM)
shCtrl
shPKM2
IC50 (μM)
Control
PKM2 KD
ECF
0.01333
0.003538
f
Survival Fraction
Time (days) after 10nM CPT-11
shCtrl
shPKM2
shPKM2+ WT PKM2
shPKM2+ T328A PKM2
g
Survival Fraction
[H2O2] (μM)
shCtrl
shPKM2
shPKM2 + WT PKM2
shPKM2 + T328A PKM2

Figure S8. a Representative western blot illustrating expression of endogenous PKM2 and exogenous flag-tagged WT, T328A, and T328E PKM2 in U87 cells used for survival analyses in Figures 6A, 6B and 6D and Supplemental Figures 6C-G. Neo, neomycin vector. **b** Representative western blot showing expression of endogenous CtIP and exogenous flag-tagged WT and T126E CtIP in cells used for survival analyses in Figure 6C. **c** U251 shCtrl or shPKM2 cells, and shPKM2 cells expressing shRNA-resistant WT, T328A, or T328E PKM2 were incubated with the indicated concentrations of olaparib. Cell viability was determined by trypan blue staining 48 h after treatment. Data are the relative mean fraction of surviving cells ± SE (n = 3). **d** U87 shCtrl or shPKM2 cells, and shPKM2 cells expressing shRNA-resistant WT or T328A PKM2, were irradiated as indicated. The number of viable cells was determined by trypan blue 5 d later. Data are presented as the relative mean fraction of surviving cells ± SE (n = 3). **e** U87 shCtrl and shPKM2 cells were incubated with the indicated concentrations of CPT-11. Cell viability was determined 72h later and used to generate the IC_{50} for each cell line. **f** U87 shCtrl and shPKM2 cells, and shPKM2 cells expressing shRNA-resistant WT or T328A PKM2, were treated with 10 nM CPT-11. The number of viable cells was determined by trypan blue staining at the indicated time points. Data are the relative mean fraction of surviving cells ± SE (n = 3). **g** U87 shCtrl or shPKM2 cells, and shPKM2 cells expressing shRNA-resistant WT or T328A PKM2, were treated with H_2O_2 as indicated. Cell viability was determined 72h later. *, P <0.05.
