# Supplementary material for: Pyruvate kinase M2 regulates homologous recombination-mediated DNA double-strand break repair
Source: Cell Res. 2018 Oct 8;28(11):1090–102. doi: 10.1038/s41422-018-0086-7 (PMC6218445; doi:10.1038/s41422-018-0086-7)
Supplement: Supplementary file 9 — Supplementary information, Figure S9 [file 41422_2018_86_MOESM9_ESM.pdf]

**a**

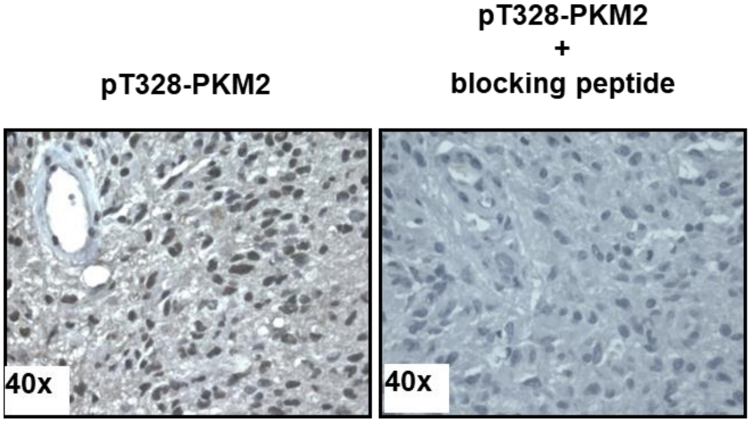


**b**

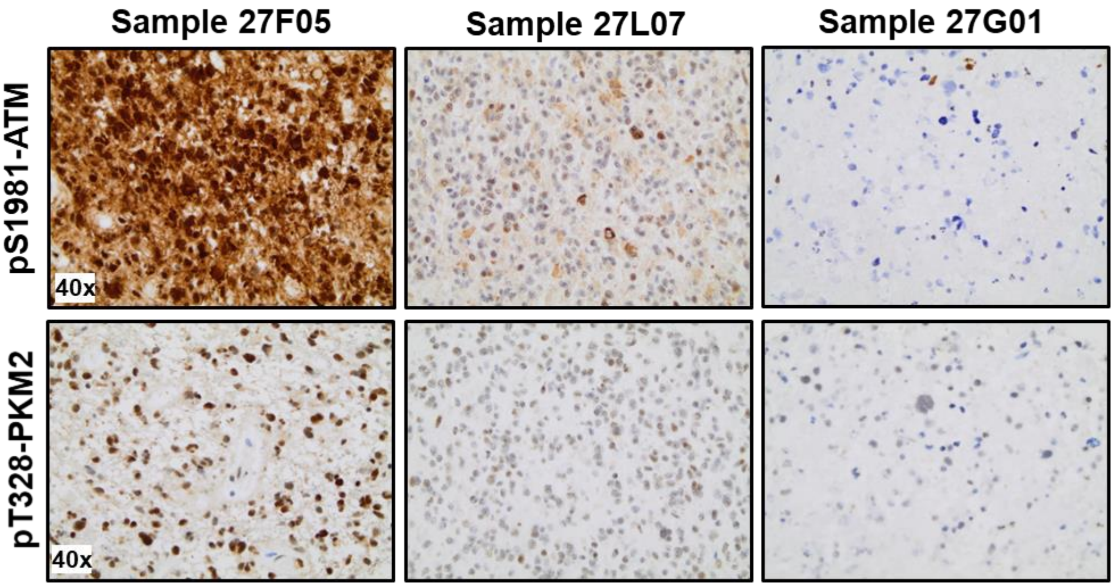


**Figure S9. a** Representative 40x images from a GBM patient sample stained with pT328-PKM2 antibody (left panel) or pT328-PKM2 antibody that was pre-incubated with phosphorylated blocking peptide. **b** Representative 40x images from three GBM patient samples illustrating the significant correlation ($P$ = 0.005) between pS1981-ATM staining (top panels) and nuclear pT328-PKM2 staining.
