# Supplementary material for: Pyruvate kinase M2 regulates homologous recombination-mediated DNA double-strand break repair
Source: Cell Res. 2018 Oct 8;28(11):1090–102. doi: 10.1038/s41422-018-0086-7 (PMC6218445; doi:10.1038/s41422-018-0086-7)
Supplement: Supplementary file 10 — Supplementary information, Data S1 [file 41422_2018_86_MOESM10_ESM.pdf]

Supplemental Methods

Cell culture

Human U87/EGFR (referred to in the text as U87) and U251 GBM cell lines were provided by Dr. Zhimin Lu (The University of Texas MD Anderson Cancer Center). T98G cells were purchased from ATCC. U87/EGFR cells in which PKM2 is stably knocked down (U87 shPKM2) along with scrambled control knockdown cells (U87 shCtrl) were also provided by Dr. Lu and have been described previously.[1] U87/EGFRvIII cells were provided by Dr. Balveen Kaur and were used to generate stable shRNA control and PKM2 knockdown cells as described previously.[1] HT1904 cells were a gift from Dr. Robert Hromas (University of New Mexico Cancer Center). U87 DR-GFP cells were generated by stable transfection of DR-GFP plasmid.[2] pEJ-H1299 cells were provided by Dr. Henning Willers (Department of Radiation Oncology, Massachusetts General Hospital, Harvard Medical School). All cells were maintained in Dulbeccos's modified Eagle's medium (DMEM) supplemented with 10% fetal bovine serum (FBS, Sigma) and 1% penicillin/streptomycin/glutamine and incubated at 37 °C with 5% CO_2. Normal human astrocytes were purchased from Lonza an grown in astrocyte growth medium as per the manusfature

DNA constructs, shRNAs, siRNAs, and transfection

His-tagged wild-type (WT) PKM2 and R399E-PKM2 were kindly provided by Dr. Zhi-Ren Liu (Georgia State University). Flag-tagged CtIP was provided by Dr. Richard Baer (Columbia University Medical Center), while HA-tagged WT-CtIP and S327A-CtIP were provided by Dr. Xiaohua Wu (The Scripps Research Institute). GST-tagged CtIP fragments were from Dr. Stephen P. Jackson (University of Cambridge). All PKM2 and

CtIP mutants were made using the QuickChange II Site-directed Mutagenesis kit (Agilent) and verified by DNA sequencing.

Non-targeting control shRNA (5’-GCTTCTAACCGGAGGAGGTCTT-3’) and shPKM2 (5’-CTATCATCTTCAATTATTTG-3’) sequences were provided by Dr. Zhimin Lu and used to produce lentivirus. Non-targeting control siRNA (CUUACGCUGAGUACUUCGAUU), and siRNAs targeting PKM1 (GCGUGGAGGCUUCUUAUAAUU), PKM2 (CCAUAAUCGUCCUCACCAAUU), CtIP (GCUAAAACAGGAACGAATCdTdT), and KU80 (GCGAGUAACCAGCUCAUAAdTdT) were purchased from Dharmacon. Transfection of siRNAs was performed using RNAiMax transfection reagent (ThermoFisher), while transfection of plasmid constructs was achieved using Fugene HD (Promega).

Western blot analysis, immunoprecipitation, cell fractionation, and antibodies

Western blots were performed as described previously.[3] Immunoprecipitation of cell lysates was performed with rabbit monoclonal or polyclonal antibodies to PKM2 (Cell Signaling), ATM (Cell Signaling), CtIP (Active Motif), or Flag (Sigma) as described in the text and figure legends. For immunoprecipitations following micrococcal nucleus digestion, samples were resuspeded in 30uL 2X reaction buffer and incubated with 320U of nuclease (New England Biolabs) at 37°C for 30m prior to immunoprecipitation. Isolation of nuclear and cytoplasmic protein fractions was achieved using a Nuclear Extract Kit (Active Motif).

Antibodies to PKM2 (Cell Signaling), PKM1 (Cell Signaling), ATM (Cell Signaling), CtIP (Active Motif), α-tubulin (Cell Signaling), Histone H1 (Cell Signaling), Flag (Sigma),

Brca1 (Millipore), Mre11 (Cell Signaling), RAD51 (Cell Signaling), RPA (Cell Signaling), GAPDH (Abcam), pT847-CtIP (Novus Biologicals) and β-actin (Cell Signaling) were used in western blot analyses as indicated.

Immunohistochemistry

Mouse brain sections bearing U87/EGFRvIII tumors, normal human brain sections (US BioMax), and human GBM sections were stained for PKM2 and PKM1 using rabbit monoclonal antibodies (Cell Signaling) at 1:800 and 1:600 dilutions, respectively. Antigen retrieval was performed with citrate buffer (pH 6.0), and the Peroxidase Histostain-Plus Kit (Invitrogen) was used per the manufacturer's protocol.

Immunofluorescence

To observe PKM2 and PKM1 subcellular localization, cells were plated on glass coverslips and subjected to treatment with ionizing radiation or EGF as described in the text and figure legends. Cells were subsequently fixed with 4% paraformaldehyde and incubated with rabbit anti-PKM2 (Cell Signaling, 1:400), rabbit anti-PKM1 (Cell Signaling, 1:400) or mouse anti-Flag monoclonal antibody (Sigma, 1:5000) as indicated. Immunofluorescence was visualized on an Olympus FV1000 confocal microscope.

I-SceI adenovirus

Adenovirus expressing I-SceI was propagated in AD293 cells as previously described.[4] Cells were exposed to the I-SceI adenovirus at an MOI of 1,000 for 3 h and then washed three times in media to remove the adenovirus.

CtIP chromatin immunoprecipitation

Primers targeting sites 604, 152, and 50 bp upstream from the I-SceI site:

50 Forward	F-5'-TCGCCGAGATCGGCCCGCGCA-3'
50 Reverse	R-5'-TTGCGGGGCGCGGAGGTCTCCA-3'
152 Forward	F-5'-TACGCACCCTCGCCGCCGCGT-3'
152 Reverse	R-5'-TGCGCGGGCCGATCTCGGCGA-3'
650 Forward	F-5'-AGCAGCCCCGCTGGCACTTGGCGC-3'
650 Reverse	R-5'-CTCAGCGGTGCTGTCCATCTGCAC-3'

Mass spectrometry analysis

CtIP bands from Flag-CtIP overexpressing U87/sh-Control treated with irradiation, U87/sh-Control without irradiation, U87/sh-PKM2 treated with irradiation, and U87/sh-PKM2 without irradiation were treated sequentially with dithiothreitol and iodoacetamide for cysteine reduction and alkylation, and then digested with modified MS-grade trypsin (Thermo Pierce) at an enzyme/substrate ratio of 1:100 in 50 mM NH_4HCO_3 (pH 8.5) at 37°C for overnight. The resulting solution was subsequently dried in a Speed-Vac. The dried residues were dissolved in water and the peptides were subsequently desalted by employing OMIX C18 pipet tips (Agilent Technologies, Santa Clara, CA) and subjected to LC-MS and LC-MS/MS analyses.

LC-MS/MS analysis in the data-dependent acquisition (DDA) mode was conducted on a Q Exactive Plus quadrupole-Orbitrap mass spectrometer (Thermo Fisher Scientific). The peptide samples were automatically loaded from a 48-well microplate autosampler using an EASY-nLC 1200 system (Thermo Fisher Scientific) at 3 µL/min onto a biphasic precolumn (150 µm i.d.) comprised of a 3.5-cm column packed with 5 µm C18 120 Å reversed-phase material (ReproSil-Pur 120 C18-AQ, Dr. Maisch). The biphasic trapping column was connected to a 20-cm fused-silica analytical column (PicoTip Emitter, New Objective, 75 µm i.d.) with 3 µm C18 beads (ReproSil-Pur 120 C18-AQ, Dr. Maisch). The peptides were then separated using a 180-min linear gradient of 6-40% acetonitrile in 0.1% formic acid and at a flow rate of 300 nL/min. The top 20 most abundant ions observed in MS were sequentially selected for fragmentation to acquire the MS/MS,

where the precursor ions were collisionally activated in the HCD cell with a normalized collision energy of 29.

Maxquant, Version 1.5.2.8, was used to analyze the LC-MS and MS/MS data for protein identification. The maximum number of miss-cleavages for trypsin was two per peptide. Cysteine carbamidomethylation was set as a fixed modification. Methionine oxidation and serine, threonine and tyrosine phosphorylation were set as variable modifications. The tolerance in mass accuracy was 20 ppm for MS and MS/MS acquired on the Q Exactive Plus mass spectrometer. The maximum false discovery rates (FDRs) were set to 0.01 at both peptide and protein levels, and the minimum required peptide length was six amino acids.

LC-MS/MS in the MRM mode were carried out on a TSQ Vantage triple quadrupole mass spectrometer equipped with a nanoelectrospray ionization source coupled to an EASY-nLC II system (Thermo Scientific). Samples were automatically loaded onto a 4 cm trapping column (150 µm i.d.) packed with 5 µm 120 Å reversed phase C18 material (ReproSil-Pur 120 C18-AQ, Dr. Maisch) at 3 µl/min. The trapping column was coupled to a 20 cm fused silica analytical column (PicoTip Emitter, New Objective, 75 µm i.d.) with 3 µm C18 beads (ReproSil-Pur 120 C18-AQ, Dr. Maisch). The peptides were then separated using a 127-min linear gradient of 4-35% acetonitrile in 0.1% formic acid and at a flow rate of 230 nL/min. The spray voltage was 1.8 kV. Q1 and Q3 resolutions were 0.7 Da and the cycle time was 4 s.

RPA and BrdU Staining

U87 cells were treated with 50uM siRNA to PKM2 (5’-CCATAATCGTCCTCACCAA-3’) or CtIP (5’-GCTAAAACAGGAACGAAT-3’) and cultured on sterile glass slides for 24h prior to further treatment. For RPA foci staining, cells were treated with DMSO or CPT-11 (2µM) for 1h. For BrdU staining cells were treated pretreated with BrdU (10mg/mL) for 16h prior to treatment with DMSO or CPT-11 (2µM) for 1h. After the specified time point, cells were stained with mouse anti-BrdU Clone B44 (BD Bioscience) or rabbit

phospho-RPA32 (S4/S8) (BENTHYL) using 1:500 dilutions. Anti-rabbit Alexa488 or anti-mouse Alexa594 conjugated secondary antibodies (Invitrogen) were used at dilutions of 1:1000. Total cells were counted under a fluorescent microscope (original magnification, ×40; Carl Zeiss, Thornwood, NY), and cells containing with greater than 10 foci were scored for positive. At least 500 cells were counted.

PKM2 kinase assay

Bacterially expressed His-tagged WT-PKM2 or R399E-PKM2 (10 μg/ml) was incubated with bacterially expressed GST-CtIP fragments (10 μg/ml) with kinase buffer (50 mM Tris-HCl [pH 7.5], 100 mM KCl, 50 mM $MgCl_2$, 1 mM Na_3VO_4, 1 mM PMSF, and 1 mM DTT) in the presence of 0.1 mM PEP or ATP in 100 μl at RT for 1h. The reactions were terminated by addition of SDS-PAGE loading buffer and heated to 100°C. The reaction mixtures were then subjected to 10% SDS-PAGE analyses and CtIP phosphorylation was detected by using a pan-phosphothreonine antibody (Cell Signaling).

ATM kinase assays

To measure phosphorylation of PKM2 by ATM *in vitro,* FLAG-tagged ATM and kinase dead mutant ATM were transiently transfected in 293T cells and immunoprecipitated using anti-FLAG M2-agarose beads (Sigma). Immunoprecipitates were extensively washed then incubated with 5μg of purified GST-PKM2 or GST-T328A PKM2 in the presence of 10μCi of [γ-^{32}P]ATP in ATM kinase buffer (25mM HEPES, pH7.4, 50mM NaCl, 10mM $MnCl_2$, 10mM $MgCl_2$,1mM DTT, 5μM ATP). The kinase reaction was conducted at 30°C for 30m. Proteins were separated by SDS-PAGE and radiolabeled proteins were visualized using a Typhoon 9410 (GE Healthcare).

To measure the phosphorylation of PKM2 by ATM *in vivo,* His-tagged PKM2 and T328A PKM2 were transiently expressed in 293T cells. 4h prior to radiation treatment, cells were washed 2x with serum-free, phosphate-free medium then 10µCi of [γ-^{32}P]ATP was added to the culture media. As needed, 20µM KU55933 was added 1h prior to treatment with 6Gy radiation. 1h after radiation, cells were lysed and PKM2 was immunoprecipitated using His-tag antibody (Cell Signaling). The immunoprecipitates were washed 3x with cell lysis buffer then resolved by SDS-PAGE. Radiolabeled proteins were detected using a Typhoon 9410 (GE Healthcare).

DNA end-resection assay

HT1904 cells containing the I-SceI DSB system were utilized to analyze DNA end-resection adjacent to I-SceI-induced DSBs as previously described.[5] Briefly, the level of resection adjacent to specific I-Sce-induced DSBs was measured by quantitative real-time polymerase chain reaction (qRT-PCR). Genomic DNA (gDNA) was extracted from HT1904 cells harvested 4 h after infection with adenoviruses expressing either I-SceI or GFP as control. Purified DNA was subjected to overnight digestion with *Xma*I (New England Biolabs) or incubated in buffer without enzyme. Digested or undigested gDNA was then used as template for qRT-PCR along with 0.5 µM of forward (5'-CGACCTTCCATGACCGAGTACAA-3') and reverse (5'-TCCGGGTCGACGGTGTG-3') primer, 0.2 µM probe (5'- 6FAMACCGCGACGACGTCCCCCGGGCC-TAMRA-3'), and 1X Taqman universal master mix (ABI). qRT-PCR was performed on a 7900HT Fast Real-Time PCR System (ABI) under standard thermal cycling conditions. The percentage of ssDNA (ssDNA%) was calculated with the following equation: ssDNA% = 1/(2^(ΔCt-1)+0.5)*100 where ΔCt was calculated by subtracting the Ct value of the undigested

sample from the CT value of the *Xma*I-digested sample. All Ct values were corrected for input DNA concentration as determined by qPCR of an amplicon on chromosome 22 containing no *Xma*I sites.

GBM patient tissue microarray

Tissue microarrays containing GBM samples in triplicate from 179 patients were provided by Dr. Pierre A. Robe (Department of Neurology and Neurosurgery, Rudolf Magnus Brain Institute, University Medical Center of Utrecht, the Netherlands). Slides were stained with antibodies to pS1981-ATM (Abcam) at a dilution of 1:200 or a custom pT328-PKM2 rabbit polyclonal antibody raised against the sequence KPVISApTQMLES at a dilution of 1:800. Staining intensity was scored by means of a Vectra 3.0 imaging system (PerkinElmer). Correlation between total pS191-ATM and nuclear pT328-PKM2 staining was conducted on data from the 66 samples for which both scores were available. Survival analysis was performed utilizing data for the 104 patients for which nuclear pT328-PKM2 (highest score from each triplicate sample was used) staining intensities and overall survival data were available.

References

1. Yang, W. et al. ERK1/2-dependent phosphorylation and nuclear translocation of PKM2 promotes the Warburg effect. *Nat. Cell Biol.* **12**, 1295–1304 (2012).

2. Golding, S. E., Rosenberg, E., Neill, S., Dent, P., Povirk, L. F. & Valerie K. Extracellular signal-related kinase positively regulates ataxia telangiectasia mutated, homologous recombination repair, and the DNA damage response. *Cancer Res.* **67**, 1046–1053 (2007).

3. Feng, Z., Kachnic, L., Zhang, J., Powell, S. N., Xia, F. DNA damage induces p53-dependent BRCA1 nuclear export. *J. Biol. Chem.* **279**, 28574–28584 (2004).

4. Fnu, S. et al. Methylation of histone H3 lysine 36 enhances DNA repair by nonhomologous end-joining. *Proc. Natl. Acad. Sci. U. S. A.* **108**, 540–545 (2011).

5. Wu, Y. et al. EEPD1 Rescues stressed replication forks and maintains genome stability by promoting end resection and homologous recombination repair. *PLoS. Genet.* **11**, e1005675 (2015).
